# Fibroblast growth factor signals drive the metastatic behavior in small cell lung cancer

**DOI:** 10.1038/s41416-025-03276-y

**Published:** 2025-12-13

**Authors:** Büsra Ernhofer, Anna Solta, Julia Sinner, Zsolt Megyesfalvi, Abigail J. Deloria, Kristiina Boettiger, Lisa Glatt, Lilla Horvath, Caterina Sturtzel, Andrea Wenninger-Weinzierl, Martin Distel, Michael Grusch, Beata Szeitz, Melinda Rezeli, Clemens Aigner, Balazs Dome, Karin Schelch

**Affiliations:** 1 Department of Thoracic Surgery, Comprehensive Cancer Center, Medical University of Vienna, Vienna, Austria; 2 Comprehensive Center for Chest Diseases, Medical University of Vienna, Vienna, Austria; 3 National Koranyi Institute of Pulmonology, Budapest, Hungary; 4 Department of Thoracic Surgery, Semmelweis University and National Institute of Oncology, Budapest, Hungary; 5 St. Anna Children’s Cancer Research Institute, Innovative Cancer Models, Vienna, Austria; 6 St. Anna Children’s Cancer Research Institute, Zebrafish Platform Austria for Preclinical Drug Screening, (ZANDR), Vienna, Austria; 7 Division of Pediatric Hematology and Oncology, Intermountain Primary Children’s Hospital, Huntsman Cancer Institute, Spencer Fox Eccles School of Medicine at the University of Utah, Salt Lake City, UT USA; 8 Center for Cancer Research, Medical University of Vienna, Vienna, Austria; 9 Department of Biomedical Engineering, Lund University, Lund, Sweden; 10 BioMS – Swedish National Infrastructure for Biological Mass Spectrometry, Lund University, Lund, Sweden; 11 Department of Translational Medicine, Lund University, Lund, Sweden

**Keywords:** Metastasis, Angiogenesis, Metastasis

## Abstract

**Background:**

Early metastatic spread represents a challenge in fighting small cell lung cancer (SCLC). The molecular mechanisms underlying metastatic dissemination remain unclear in this devastating disease.

**Methods:**

Invasive traits were investigated in 13 SCLC cell lines using 3D-spheroid formation, sprouting assays, co-cultures and a zebrafish xenograft model. Proteomic analysis was performed to unravel metastatic drivers, which were validated by qPCR, growth factor arrays and specific inhibitors.

**Results:**

Overall, 8 cell lines formed spheroids, and half of these displayed invasive sprouting in collagen. The ‘sprouter’ SCLC cells, which all had a YAP1-dominant subtype, showed increased migration in zebrafish larvae and penetrated endothelial cell monolayers to a higher extent, thereby mimicking intra- and extravasation. Proteomics revealed differences in adhesion properties, oncogenic pathways and receptor tyrosine kinase signalling. Sprouter cells showed higher expression levels of mesenchymal cell state markers. Stimulation with fibroblast growth factor 2 (FGF2) further induced invasive sprouting, while blocking the FGF/R axis resulted in a significant reduction of sprouting in vitro and in vivo.

**Conclusion:**

The FGF/R axis is a key driver of SCLC metastatic spread in the YAP1-dominant subtype. These data might facilitate the development of potential future therapies targeting FGF/R signalling to prevent SCLC progression and metastasis.

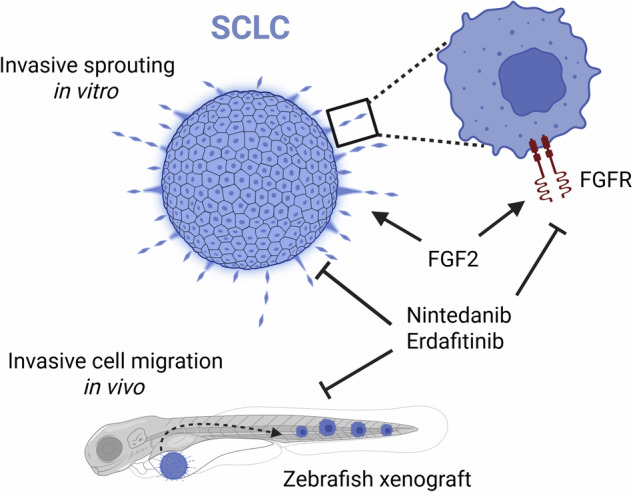

## Introduction

Approximately 15% of lung cancer cases are classified as small cell lung cancer (SCLC), which is characterised by aggressive growth, acquired resistance and poor prognosis [1]. One major impediment in treating SCLC is the frequent occurrence of distant metastases, which are often present at diagnosis or emerge early during disease progression [2]. Overall, SCLC patients commonly present liver and adrenal gland metastases, whereas brain, bone, or pericardial lesions tend to appear in later stages [3]. Despite advances in chemo(-radio)therapy and immunotherapy, SCLC therapeutic options remain limited due to systemic spread and acquired therapy resistance. Current standard-of-care regimen, typically involve a platinum-based agent combined with etoposide, but are often inadequate in controlling metastatic disease. Although recent molecular classifications have identified four distinct molecular subtypes predominantly driven by specific transcription factors including, achaete-scute homologue 1 (ASCL1), neurogenic differentiation factor 1 (NEUROD1), POU class 2 homeobox 3 (POU2F3) and yes-associated protein 1 (YAP1), these insights have yet to be translated into improvements in therapeutic outcomes [4]. The latter subgroup has recently been described as an inflamed phenotype with inflammatory characteristics [5]. Each of these subtypes (furthermore SCLC-A/N/P/Y) were previously characterised using mass spectrometry-based proteomics, highlighting unique proteomic signatures associated with each subtype [6]. While molecular subtyping of SCLC has offered molecular insights, the frequent occurrence of metastases remains a major problem, driving the need for treatments that can specifically target these metastatic pathways.

Comparative proteomic analyses of the four SCLC subtypes revealed that SCLC-Y was highly associated with epithelial-mesenchymal transition (EMT) indicating an increased potential for SCLC metastasis [6]. EMT is a biological process in which epithelial cells transform into mesenchymal cells by acquiring morphologic and behavioural features of mesenchymal cells [7]. Growth factors, particularly those of the fibroblast growth factor/receptor (FGF/R) axis, play a crucial role in regulating EMT and promoting cancer cell invasion and metastasis. This process is central to tumour progression as it equips cancer cells with the enhanced migratory ability to invade surrounding tissues [7].

Since little is known about the mechanistic background of early metastatic spread in SCLC, in this study, we aimed to characterise the invasive capacity of SCLC cells using 3D-approaches and a zebrafish xenograft model as well as identify novel approaches to target metastatic dissemination, which is crucial for developing new therapies for SCLC patients.

## Materials and methods

### Cell culture

A total of 13 human SCLC cell lines, GFP-expressing immortalised blood endothelial cells (BEC) and lymphatic endothelial cells (LEC) as well as HEK293 cells were used in this study. All cells were provided by collaboration partners or purchased from the American Type Culture Collection (ATCC), authenticated by STR profiling in 2024 and have been previously described by our group [6, 8]. Endothelial cells were maintained in EGM-2 MV Endothelial medium (Lonza, Basel, Switzerland) and SCLC and HEK293 cells were cultivated in RPMI 1640 with L-glutamine and DMEM medium (Sigma, St. Louis, MO, USA), respectively, supplemented with 10% heat-inactivated foetal calf serum (FCS) in a humidified atmosphere (5% CO_2_, 37 °C). The cultures were regularly tested for *Mycoplasma* contamination. Information about cell lines is summarised in Supplementary Table S1.

### Generation of transgenic cell lines

Six SCLC cell lines were infected with retroviral particles to stably express turbo Red Fluorescent Protein (RFP). Viral constructs were produced in HEK293 cells by calcium phosphate co-precipitation-mediated co-transfection of the plasmids pRXTOP-RFP, VSV-G, and pgag-pol-gpt as previously described [8, 9]. After 24 h of incubation, the virus-containing medium was replaced by fresh RPMI (10% FCS) and cells were selected using puromycin (MedChemExpress, Monmouth Junction, NJ, USA) at a concentration of 0.8 μg/ml.

### Drug treatment

To inhibit specific intracellular pathways, we treated the cells with a panel of small-molecule inhibitors, dissolved in DMSO. In brief, we used nintedanib, erlotinib, erdafitinib, galunisertib, GSK2816126A, KRN-633, K-975, SB-431542, seralutinib, XMU-MP-1 and Wnt-C59 (all obtained from MedChemExpress). A list of the used inhibitors and their molecular targets is provided in Supplementary Table S2. For cell viability assays, concentrations from 1 µM to 20 µM were used. For sprouting assays, all inhibitors were used at a concentration of 10 µM, except for nintedanib (0.5 µM), erdafitinib (0.5 µM) and XMU-MP-1 (1 µM). For western blots, erdafitinib was used at 10 µM. Recombinant FGF2 was purchased from Thermo Fisher Scientific (Waltham, MA, USA) and used at 10 ng/ml and 50 ng/ml for sprouting assays and at 10 ng/ml for western blots.

### Cell viability assay

Cells (5 × 10^3^) were seeded in a flat-bottom 96-well plate, incubated overnight and then treated for 96 h with the inhibitors or vehicle (DMSO) at the indicated doses. Cell viability was assessed using EZ4U (Biomedica, Vienna, Austria) according to the manufacturer´s instructions. The absorbance was determined at 450 nm and 620 nm wavelengths using a Varioskan Lux microplate reader (Thermo Fisher Scientific).

### Spheroid formation assay

After cell counting, SCLC cells (5 × 10^3^) were seeded in medium containing 20% methyl cellulose (Sigma) in a 96-well U-bottom suspension plate (Greiner Bio-One, Kremsmünster, Austria). The plates were subsequently centrifuged at 2000 × *g* for 5 min at room temperature (RT) and incubated at 37 °C for 48 h to allow spheroids formation.

### 3D invasion/sprouting assay

For spheroid sprouting assays, previously generated spheroids were embedded in a collagen matrix into an agarose-coated plate as previously described [10]. Briefly, collagen (1 mg/ml final concentration) was prepared according to the company´s instructions (Collagen Type I Rat Tail; Corning). Meanwhile, a 96-well plate (F-bottom) (Greiner Bio-One) was coated with 1% low melting point agarose (LMP ultrapure; Life Technologies, Carlsbad, CA, USA) and stored at 4 °C. Spheroids were aspirated in collagen medium and transferred into the agarose-coated 96-well plate. After the collagen solidified (1 h, 37 °C), growth medium with or without treatment was added as indicated. For inhibitor treatment, spheroids were embedded in medium with 10% FCS, assays using recombinant FGF2 were performed in medium containing 2.5% FBS. Images were acquired 0 h, 24 h, 96 h and 120 h after embedding using an inverse microscope (Micro Ti Eclipse FL, Nikon, Minato City, Tokyo, Japan or Axiovert 40c, Carl Zeiss, Oberkochen, Germany) and manually evaluated using the ImageJ 1.54 software (Wayne Rasband, National Institutes of Health, USA) [11].

### CCID formation assay

To assess the formation of circular chemorepellent-induced defects (CCID), SCLC spheroids were co-cultured with endothelial cells as described [8]. Briefly, GFP-expressing, immortalised LEC and BEC were harvested and cells (1.5 × 10^4^) were seeded in 96-well plates. After 48 h, previously generated SCLC spheroids were placed onto the endothelial cell monolayer. Images were acquired after 7 h, 24 h and 32 h using an inverse Micro Ti Eclipse FL microscope. Analyses were performed using ImageJ.

### RNA isolation and qPCR

Spheroids were generated as described above and incubated for 72 h. Spheroids were collected in 1 ml TRIZOL (Gibco, Grand Island, NY, USA) and RNA isolation was conducted using chloroform and isopropanol. Total RNA was measured using the Nanodrop 8000 Spectrophotometer (VWR catalyst). Subsequent cDNA synthesis was performed using the High capacity cDNA reverse transcription kit (Thermo Fisher Scientific). SYBRgreen based (SYBR Green qPCR Master Mix Universal, Medchemexpress) and TaqMan based (TaqMan®Gene Expression Master Mix, Life Technologies) approaches were used for qPCR. The following primers were used for SYBRgreen-based assays: vimentin (fwd GGCTCAGATTCAGGAACAGC, rev CTGAATCTCATCCTGCAGGC) twist (fwd CGACGAGCTGGACTCCAAGATG, rev AGACCGAGAAGGCGTAGCTG), snail (fwd TATGCTGCCTTCCCAGGCTTG, rev ATGTGCATCTTGAGGGCACCC), MMP1 (fwd TACATGCGCACAAATCCC, rev ACAGCCCAGTACTTATTCCC), GAPDH (fwd AGCTCACTGGCATGGCCTTC, rev ACGCCTGCTTCACCACCTTC). TaqMan assay IDs used in this study comprised FGF-1, Hs01106908_m1, FGF-2, Hs00266645_m1, FGF-5, Hs00170454_m1, FGF-18, Hs00826077_m1, FGFR1, Hs00915142_m1, FGFR2, Hs01552918_m1, FGFR3, Hs00179829_m1, FGFR4 and Hs01106908_m1.

### Protein isolation and western blots

Cells (5 × 10^5^) were seeded into 6-well plates and on the next day treated as indicated. After 24 h, cells were harvested in RIPA buffer supplemented with Protease Phosphatase Inhibitor Cocktail (Thermo Fisher Scientific). Protein concentrations were determined using the Pierce BCA assay (Thermo Fisher Scientific). Lysates were separated using Mini-PROTEAN Tetra electrophoresis cells (Bio-Rad Laboratories Hercules, California, USA) and transferred onto a nitrocellulose membrane (Amersham Protran, GE Healthcare) through Trans-BlotTurbo (Bio-Rad Laboratories). Immunodetection was performed with Super Signal West Femto Chemiluminescent Substrate (Thermo Fisher Scientific) as described [12]. The following primary antibodies were used at a concentration of 1:1000 overnight at 4 °C: pYAP1 (Ser127, D9W2I, #13008, CST), CYR61 (#39382, CST), pERK1/2 (Thr202/Tyr204, D13.14.4E, #4370, CST), ERK1/2 (137F5, #4695, CST). GAPDH (#5174, CST, 1:10000) was used as a loading control and secondary antibodies were purchased from Dako and used at a dilution of 1:1000. Chemiluminescence visualised on a Vilber Fusion FX system. Band intensity quantification was performed with ImageJ.

### Zebrafish xenograft model

Transparent zebrafish (Danio rerio) mutants (mitfa^b692/b692^; ednrba^b140/b140^) with or without stably expressing GFP in the circulation system (Tg(fli1a:EGFP)^y1^) were bred and maintained at standard conditions under license GZ:565304-2014-6 of the local authorities at the CCRI zebrafish facility [13, 14]. For xenotransplantation (license GZ:333989-2020-4), zebrafish larvae at 2 days post fertilisation were anaesthetised using 1x tricaine (eq. 0.16 g/L Ethyl-3-aminobenzoat-methansulfonat (Sigma Aldrich, California, USA) with Tris-HCl (pH 9.0) from a 25x stock (pH 7.2) in E3 medium) and ~200–300 RFP-expressing SCLC cells were injected into the perivitelline space (PVS) using a FemtoJet 4i microinjector (Eppendorf, Hamburg, Germany) as previously described [15]. For engraftment tests the larvae were sorted for larvae with tumour cells only in the PVS at 2 h post injection and further cultivated at 34 °C. Then, in the treatment setting, nintedanib, erdafitinib or the respective amount of DMSO were added to the water at a concentration of 10 µM on the next day. This is the highest tolerable concentration for the larvae. Automated imaging was carried out on an Operetta CLS high-content imager (Revvity (formerly PerkinElmer), Waltham, MA, USA) 1 and 3 days post injection (dpi). To compare tumour sizes automated measurements of the tumour area based on fluorescence were conducted as previously reported [16]. For quantification of the number of cells in the tail region, images were analysed using ImageJ as described [8].

### Growth factor array

Cells (2.5–5 × 10^5^) were seeded in 6-well plates and incubated overnight. Next, cells were washed with PBS and incubated for 24 h using serum-free medium. Supernatant was collected and centrifuged at 1 × 10^4 ^rpm for 5 min. Samples were stored at −20 °C until analysed using the Human Growth Factor Array (Abcam, Cambridge, UK) according to the manufacturer´s instructions. In brief, antibody array membranes were blocked and supernatants were incubated overnight at 4 °C under gentle agitation. Membranes were incubated with biotin-conjugated anti-cytokines following HRP-conjugated streptavidin for 2 h at RT. Between each incubation step, membranes were thoroughly washed. Chemiluminescence imaging was conducted on a Vilber Fusion FX system. Spot signals were quantified using ImageJ and normalised to control spots.

### Proteomic analysis

This study includes parts of our previously published proteomic dataset of 26 human SCLC cell lines. For further details, see Szeitz et al. [6]. In brief, groups were assigned according to the invasive in vitro behaviour into ‘sprouter’ (H372, H196, H1341, HLHE) and ‘non-sprouter’ (DMS53, GLC4, H524, H378). Differential expression analyses for the proteomic data were performed via ANOVA, followed by multiple testing correction (Benjamini-Hochberg, BH) of ANOVA *p* values and Tukey’s honestly significant difference (HSD) post-hoc tests. A protein was considered significant for a comparison if both the ANOVA FDR and the corresponding pairwise Tukey’s HSD test *p* value was less than 0.05. Pathway analysis performed by 1D annotation enrichment analysis using the KEGG database in Perseus v2.0.7.0 [17]. Additionally, gene set enrichment analysis was performed using the GSEA software version 4.3.2 based on the molecular signature database (MSigDB) hallmark gene set [18, 19].

### Statistical analysis

Unless stated otherwise, statistical evaluation was performed on at least three independent biological replicates. Data was analysed in GraphPad Prism 8.0 by either the Mann-Whitney *t*-test, one-way ANOVA or Kruskal Wallis multiple comparisons test. A *p* ≤ 0.05 was considered statistically significant.

## Results

### SCLC cells exhibit different capacities to generate spheroids and invasive sprouts

**Fig. 1:**
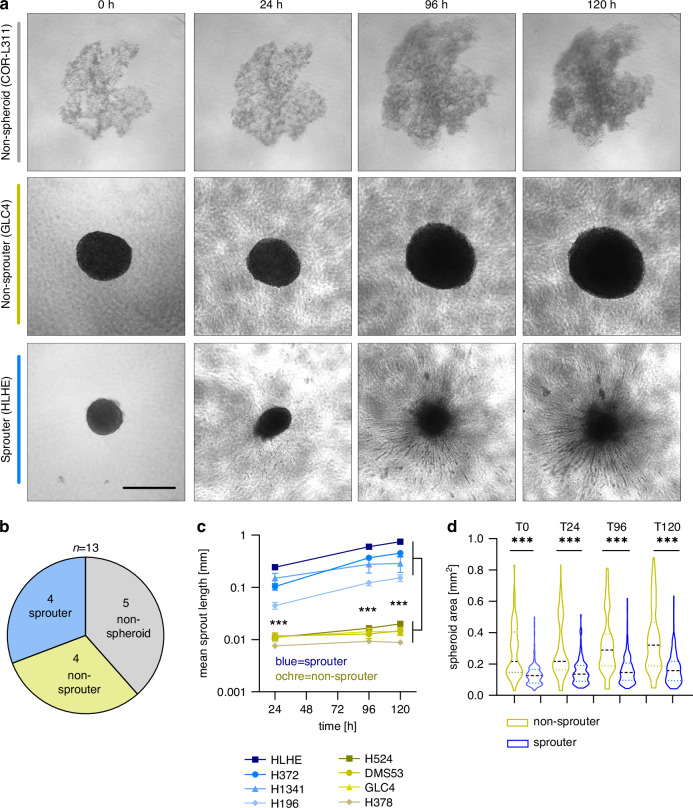
SCLC cell lines display differential invasive potential in vitro. **a** SCLC cell lines are distinguished based on spheroid formation and invasive sprouting into non-spheroid (top, grey), non-sprouter (middle, ochre) or sprouter (bottom, blue) in a time-dependent manner. Scale bar: 500 µm. **b** Pie chart displaying grouping of SCLC cell lines. **c** Quantification of the mean sprout length. At least 10 sprouts of minimum 15 individual spheroids were quantified. ANOVA and Kruskal-Wallis test. ***p < 0.001 **d** Pooled results comparing the mean area of sprouter (blue; HLHE, H372, H1341, H196) versus non-sprouter (ochre, H524, GLC4, DMS53, H378) groups. ANOVA and Kruskal-Wallis test. ***p < 0.001.

In order to assess the aggressive potential of our SCLC cell models, we tested their ability to grow in spherical structures and to form invasive sprouts when embedded in a collagen matrix. Due to the non-adherent, semi-adherent or adherent growth properties of SCLC cell lines, experiments were performed in 3D culture settings. Out of 13 cell lines, 5 grew in loose aggregates (‘non-spheroid’; H82, H1048, H1694, H2171, COR-L311), while the other 8 were able to form compact spheroids when embedded. From those, 4 cell lines showed invasive sprouting already after 24 h (‘sprouter’; H196, H372, H1341, HLHE), which increased in a linear fashion over time. The other 4 SCLC cell lines (‘non-sprouter’; H524, GLC4, DMS53, H378) remained as compact spheroids for up to 120 h (Fig. 1a, b). Quantification of the mean sprout length reflected the substantial difference between the groups (Fig. 1c). Spheroid areas minimally increased over time, with non-sprouter cell lines generally being greater in size than sprouters (Fig. 1d, Supplementary Fig. S1).

### Sprouter cell lines are more invasive in co-cultures and zebrafish

**Fig. 2:**
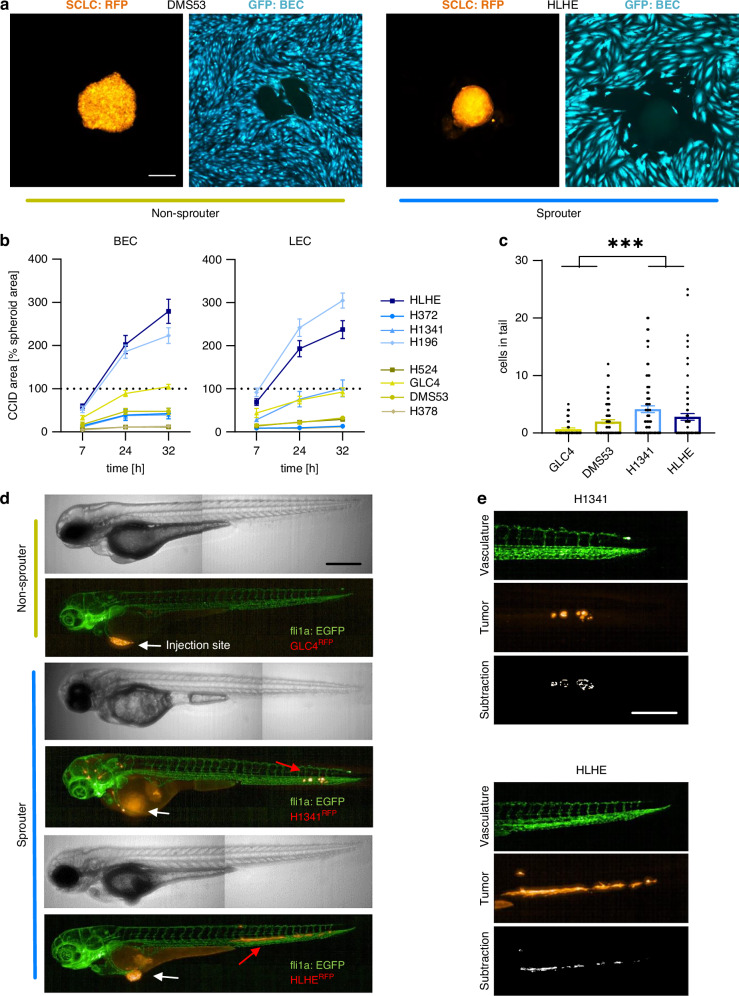
Sprouter cell lines are more invasive in co-cultures and zebrafish. **a** Representative images of RFP-expressing spheroids from non-sprouter (DMS53) and sprouter (HLHE) cells (orange) creating circular chemorepellent-induced defects (CCID) in GFP-expressing blood endothelial cell (BEC) monolayers (cyan) after 32 h of co-culturing. Scale bar: 250 µm. **b** CCID area created by SCLC spheroids in BEC (left) and LEC (right) monolayer over time (7–32 h). Data is shown as the percentage of the CCID to spheroid area (mean ± SEM) of at least 30 individual spheroids. **c** Quantitative analysis of cells in the tail region of zebrafish larvae after 24 h (manual count). Each dot represents one fish. Comparison between two non-sprouter (ochre; GLC4 and DMS53) and two sprouter (blue; H1341 and HLHE) cell lines. Statistical significance between the groups was calculated after 24 h using the one-way ANOVA and Tukey´s multiple comparisons test. ***p < 0.001 **d** Representative images of fli1a:EGFP transgenic zebrafish injected with RFP-expressing SCLC cells 1 day post injection. White arrows indicate the injection sites, red arrows indicate tumour cells in the tail. Scale bar: 250 µm. **e** Masks of single channels (GFP: vasculature, RFP: tumour cells) and corresponding subsequent subtraction showing extravascular cells (white) in representative fish. Scale bar: 50 µm.

To test the ability of inducing circular CCID, we co-incubated RFP-expressing tumour spheroids with a monolayer of GFP-expressing endothelial cells (Fig. 2a). All 8 SCLC cell lines tested were able to form gaps in both, blood (BEC) and lymphatic (LEC) endothelial cell monolayers, in a time-dependent manner, although only 2 sprouter cell lines caused gaps larger than the spheroids (Fig. 2b). However, when assigning the cell lines based on their respective sprouting behaviour, we found a significantly increased CCID formation ability in the sprouter group (Supplementary Fig. S2). Additionally, we discovered a strong correlation between CCID formation in BECs and LECs, indicating no evident preference of SCLC cells in penetrating blood or lymphatic monolayers (Supplementary Fig. S3). Next, we were interested in whether the differences in invasive properties observed in vitro were also present in vivo. To this aim, 2 representative sprouter (H1341, HLHE) and 2 non-sprouter (DMS53, GLC4) cell lines expressing RFP were injected into the perivitelline space of zebrafish larvae and the migration into the tail region after 24 h was quantified. Our results show that fish injected with the cell lines which demonstrated in vitro sprouting ability had significantly more tumour cells in the tail region (Fig. 2c, d). To confirm that tumour cells were indeed able to invade into the surrounding tissue, we created masks of single channel (vasculature, tumour cells) pictures and used subsequent subtraction to detect extravascular cells (Fig. 2e).

### Sprouter and non-sprouter cells differ in protein expression

**Fig. 3:**
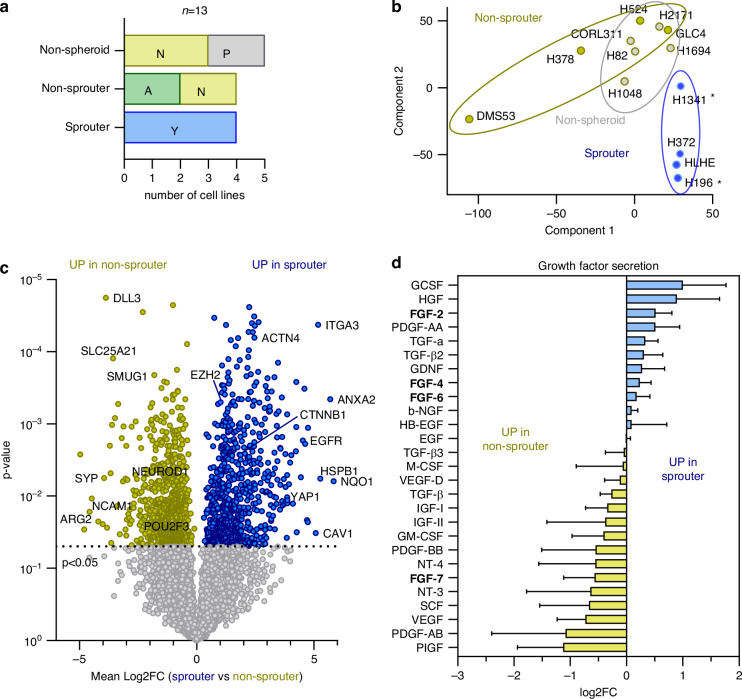
Sprouter and non-sprouter cells differ in protein expression*.* **a** Subtype distribution including A = ASCL1, N = NEUROD1, P = POU2F3, Y = YAP1 of non-spheroid, non-sprouter and sprouter groups. **b** Principal component analysis (PCA) of sprouter (blue), non-sprouter (ochre) and non-spheroid (grey) cell lines based on their protein expression profile. The asterisks indicate the cell lines mentioned by Ng et al. **c** Volcano plot depicting the differentially expressed proteins in sprouter (blue; HLHE, H196, H372, H1341) and non-sprouter cell lines (ochre; DMS53, GLC4, H378, H524). The dotted line indicates a *p* value of 0.05, which was used as cut-off to define differentially expressed proteins. **d** Growth factors present in the supernatant of non-sprouter (DMS53 and GLC4) and sprouter cell lines (HLHE and H1341), determined by growth factor arrays. The data is shown as mean log2 fold change (FC) ± SEM, calculated from the mean pixel intensities of each sprouter against each non-sprouter cell line. The positive control of each blot was used as reference.

In order to better understand the underlying mechanisms of invasion, we re-analysed the proteomic dataset from our previously published study characterising molecular SCLC subtypes [6]. Our previous study attributed invasive characteristics to cells with high YAP1 expression. In line with this, the 4 sprouter cell lines were exclusively of the YAP1-dominant subtype, whereas the non-spheroid-forming and non-sprouter cell lines had mixed SCLC-A, SCLC-N or SCLC-P molecular backgrounds (Fig. 3a). Proteomic comparison displayed apparent separation of the 4 sprouter (blue) and 4 non-sprouter (ochre) cell lines based on principal component analysis (PCA) (Fig. 3b). The 5 non-spheroid-forming cell models (grey) partly overlap with the non-sprouters. Although 2 of the YAP1-expressing sprouter cells are defined as SCLC according to the ATCC, a recent study [20] questioned the pulmonary origin of H1341 cell line and the small cell nature of H196 cells. Since in the PCA plot these cells cluster well together, we continue to refer to all 4 cell lines as ‘sprouters’ throughout the manuscript.

Examination of the differentially expressed proteins of each group revealed 741 and 825 proteins significantly overexpressed in sprouter and non-sprouter cells, respectively (Fig. 3c, Supplementary Table S3). Besides the subtype-defining YAP1, sprouter cell lines were characterised, among many others, by enhanced expression of integrin subunit alpha 3 (ITGA3), vimentin (VIM), actin 1 and 4 (ACTN1, ACTN4), and epidermal growth factor receptor (EGFR). Contrarily, the non-sprouter cell lines overexpressed neural cell adhesion molecule 1 and several members of the solute carrier family (SLCs). Also, as defined by their respective subtypes, we found predominant neuroendocrine (NE) patterns including delta-like ligand 3 (DLL3), synaptophysin (SYP) and neurogenic differentiation factor 1 (NEUROD1), as well as the POU class 2 homeobox 3 (POU2F3) characteristic for the SCLC-P subtype (Fig. 3c, Supplementary Table S3).

In addition to the intracellular proteins, we also investigated secreted growth factors. When comparing the supernatants of 2 sprouter (HLHE, H1341) and 2 non-sprouter cell lines (GLC4, DMS53), we observed enhanced levels of hepatocyte growth factor (HGF), several fibroblast growth factors (FGF2, FGF4, FGF6), granulocyte-colony stimulating factor (GCSF), platelet-derived growth factor alpha (PDGF-AA) and transforming growth factors alpha and beta (TGF-a, TGF-b) in the sprouter cells (Fig. 3d, Supplementary Fig. S4). In contrast, the non-sprouter cell lines secreted more placental growth factor (PGF), platelet-derived growth factor beta (PDGF-AB, PDGF-BB), vascular endothelial growth factor (VEGF) and insulin-like growth factors (IGF-I, IGF-II) (Fig. 3d, Supplementary Fig. S4).

### Sprouter cell lines are characterised by distinct signalling cascades and display an EMT-like phenotype

**Fig. 4:**
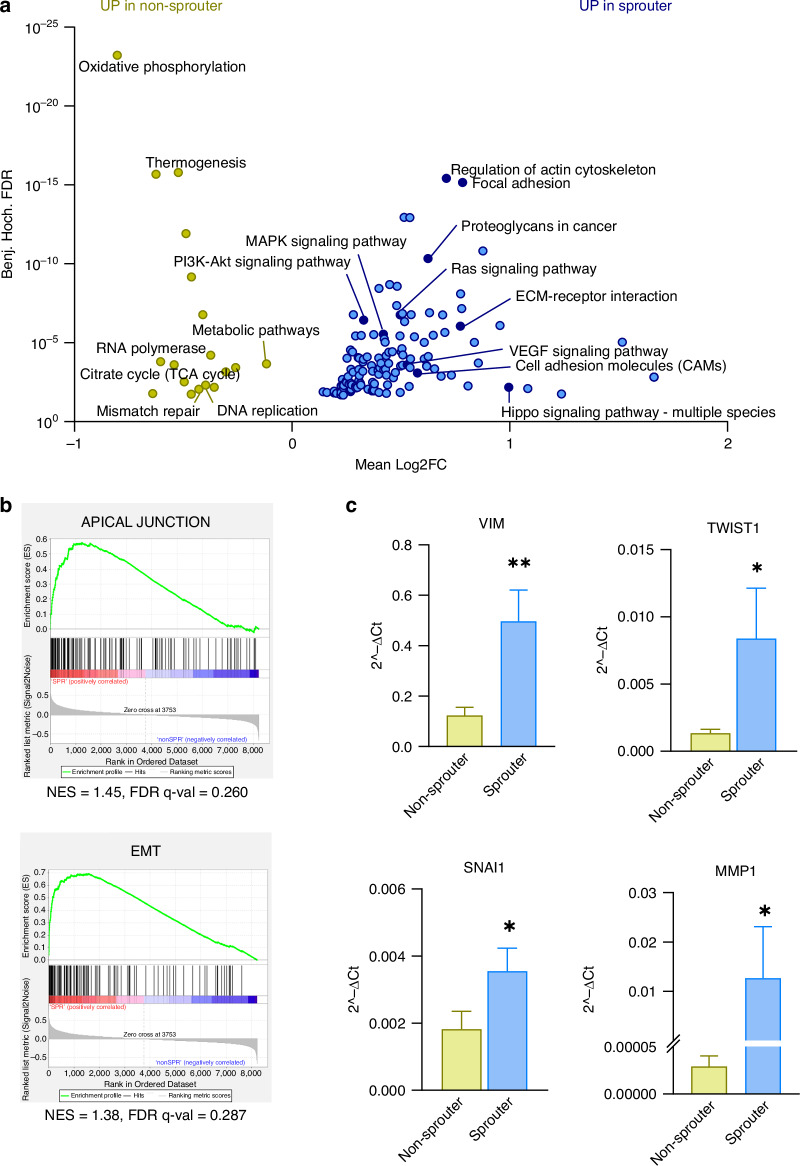
Proteomic analysis reveals dysregulated pathways involved in key signalling cascades. **a** 1D annotation enrichment between sprouter (blue; HLHE, H196, H372, H1341) and non-sprouter (ochre; DMS53, GLC4, H378, H524) cell lines based on the KEGG database. **b** Enrichment of ‘apical junction’ and ‘epithelial-to-mesenchymal transition’ (EMT) in sprouter compared to non-sprouter cell lines based on gene set enrichment analysis (GSEA v4.3.2) performed using proteomics data from sprouter and non-sprouter cell lines, considering the Hallmark dataset. NES (normalised enrichment score) and FDR (False Discovery Rate) *q* values are shown in the graphs. A FDR *q* < 0.25 is considered significant. The respective *p* values are 0.025 for ‘apical junction’ and 0.078 for ‘EMT’. **c** RNA expression analysis of key mesenchymal markers, including *VIM, TWIST1*, *SNAI1* and *MMP1* in pooled non-sprouter (ochre) and non-sprouter (blue) cell lines (*n* = 4). Data is shown as mean ± SEM. Mann-Whitney test. *p < 0.05, **p < 0.01.

Looking deeper into the distinct expression signatures between cell lines with sprouting or non-sprouting capacities, pathway analysis revealed numerous differentially enriched processes. Hence, results from 1D annotation enrichment analysis using the KEGG database revealed 18 and 130 significantly upregulated pathways in the non-sprouter and sprouter groups, respectively (Fig. 4a, Supplementary Table S4). The signature of the latter indicated increased adhesive properties by means of upregulated pathways, such as regulation of actin cytoskeleton, focal adhesion, and proteoglycans in cancer, among others. Moreover, the results comprised the involvement of major signalling cascades, including PI3K-Akt signalling, MAPK signalling, Ras signalling, Hippo signalling, and VEGF signalling. The non-sprouter cell lines displayed a metabolic shift towards oxidative phosphorylation, thermogenesis, and citrate cycle in addition to DNA repair mechanisms, including mismatch repair and DNA replication (Fig. 4a). Additional gene set enrichment analysis (GSEA) revealed an enrichment of apical junction and epithelial-to-mesenchymal transition (EMT) pathways in sprouter cell lines (Fig. 4b, Supplementary Fig. S5). Since the proteomic data was obtained from 2D cell cultures, we validated the EMT markers vimentin (*VIM*), twist-related protein 1 (*TWIST1*), snail family transcriptional repressor 1 (*SNAI1*), and matrix-metalloproteinase 1 (*MMP1*) in our 3D models and found significantly increased mRNA expression in the sprouter cell lines (Fig. 4c). To confirm the similar characteristics between the genuine and questioned SCLC cell lines suggested by Ng et al., we performed similar pathway analyses followed by pre-ranked GSEA using a different grouping. We compared the enriched pathways from the 4 non-sprouters vs. the 2 SCLC-Y cell lines (H372 and HLHE) with those from the 4 non-sprouters vs. the 2 SCLC-Y cell lines in question (H196 and H1341) (Supplementary Fig. S6A). Our results showed a 69.6% overlap between significantly enriched pathways, which we consider within the normal range of biological variation between cell models (Supplementary Fig. S6B).

### Invasive sprouting depends on the FGF axis

Next, to identify the molecular pathway driving the invasive behaviour in our sprouter cells, we assessed their ability to form invasive sprouts after treatment with a variety of specific inhibitors at sublethal concentrations. Our inhibitor panel included the FGFR inhibitor erdafitinib, EGFR inhibitor erlotinib, TGFβR inhibitors galunisertib and SB-431542, EZH2 inhibitor GSK2816126A (GSK126), VEGFR1-3 inhibitor KRN-633, YAP1/TAZ and TEAD inhibitor K-975, VEGFR1-3, FGFR1-3, PDGFR inhibitor nintedanib (pan inhibitor), PDGFRα, β inhibitor seralutinib, MST1/2 inhibitor XMU-MP-1, and PORCN inhibitor Wnt-C59.

**Fig. 5:**
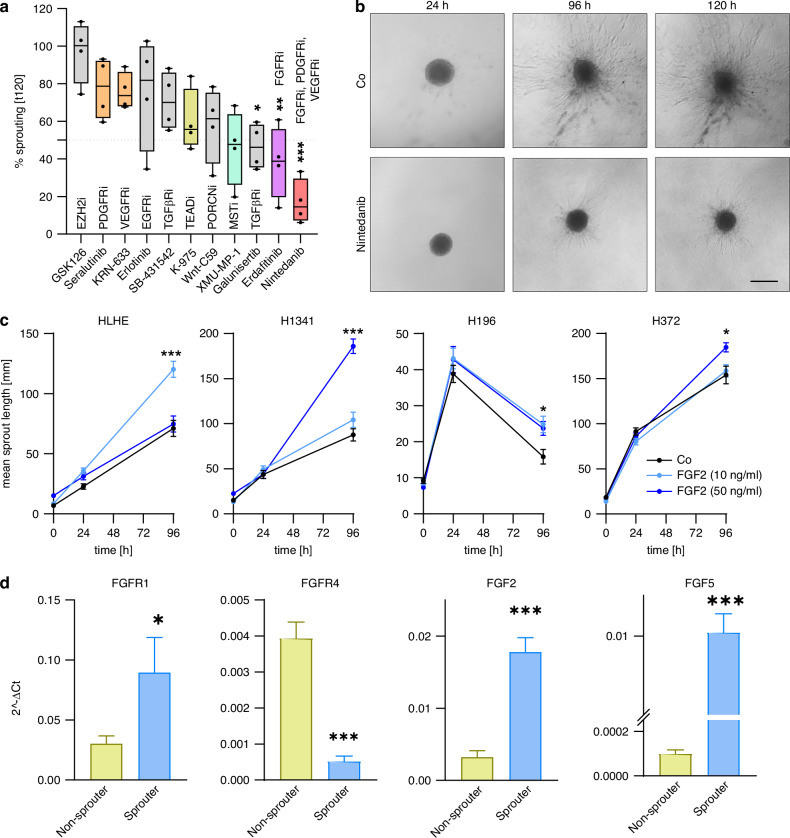
Sprouting potential is reduced after FGFR inhibition. **a** Pooled data of sprouting inhibition compared to DMSO controls after 120 h of sprouter cell lines (HLHE, H196, H372, H1341) after treatment with FGFR inhibitor erdafitinib, EGFR inhibitor erlotinib, TGFβR inhibitor galunisertib and SB-431542, EZH2 inhibitor GSK2816126A (GSK126), VEGFR1-3 inhibitor KRN-633, YAP1/TAZ and TEAD inhibitor K-975, VEGFR1-3, FGFR1-3, PDGFR inhibitor nintedanib, PDGFRα, β inhibitor seralutinib, MST1/2 inhibitor XMU-MP-1 and PORCN inhibitor Wnt-C59. Each dot represents one cell line. Statistical evaluation was performed using one-way ANOVA and DUNN´s multiple comparison test. *p < 0.05, **p < 0.01, ***p < 0.001 **b** Representative images of sprouting ability of HLHE cells with control (DMSO, upper panel) and nintedanib (0.5 µM, lower panel) treatment after 24 h, 96 h, and 120 h. Scale bar: 500 µm. **c** Quantification of sprouting based on spheroid sprouting assays with reduced serum (2.5% FBS). Spheroids were treated with 10 ng/ml and 50 ng/ml recombinant FGF2 for 96 h. Mean sprout length over time is shown as mean ± SEM of at least 10 individual spheroids. Two-way ANOVA and Tukey´s multiple comparison test. ***p < 0.001. **d** RNA expression of FGFRs and FGFs in pooled non-sprouter (ochre) and non-sprouter (blue) cell lines (*n* = 4), determined by qPCR. Data is shown as mean ± SEM. Mann-Whitney test. *p < 0.05, **p < 0.01, ***p < 0.001.

Sublethal concentrations (viability > 75%) for the sprouting assays were determined by MTT-based cell viability assays (Supplementary Fig. S7). Inhibitors were used at 10 µM, but nintedanib and erdafitinib were reduced to 0.5 µM and XMU-MP-1 to 1 µM due to high sensitivity. Overall, several inhibitors resulted in decreased sprouting in some cell lines, while the greatest and most consistent effects were observed when treating the cells with the multi-RTK inhibitor nintedanib (red), with more than 65% inhibition in all cell lines (Fig. 5a, b, Supplementary Fig. S8). The second strongest overall sprout inhibition was achieved with the FGFR inhibitor erdafitinib (pink), identifying the FGF/R axis as the crucial pathway. Specific inhibition of the other targets of nintedanib, VEGFR and PDGFR (orange), with KRN-633 and seralutinib, respectively, had no effect (Fig. 5a, Supplementary Fig. S8). Interestingly, although only the sprouter cell lines express YAP1, invasive sprouting was not directly affected by modulation of YAP1 activity via K-975 (ochre) and XMU-MP-1 (turquoise), which inhibit and activate YAP1 transcriptional activity, respectively (Fig. 5a, Supplementary Fig. S8). Also, we found no significant differences in sensitivity between sprouters and non-sprouters to our panel of inhibitors (Supplementary Fig. S9).

Next, to functionally validate the role of FGFR-mediated signals in aggressive SCLC behaviour, we treated spheroids with recombinant FGF2 (10 ng/ml and 50 ng/ml) and found a significant stimulation of invasive sprouting in all 4 sprouter cell lines after 96 h (Fig. 5c), while the non-sprouter cells remained largely unaffected (Supplementary Fig. S10).

These findings are also supported by the high levels of FGFs in the supernatant of sprouter cells described in Fig. 3d. Concerning mRNA expression of the FGF/R axis members in SCLC spheroids, of the four FGF receptors, FGFR1 and 2 were upregulated, while FGFR3 and 4 were downregulated in the sprouter cells compared to the non-sprouters. Among the ligands, FGF1, 2 and 5 were higher expressed by sprouters, and FGF18 was slightly more abundant in non-sprouter cell lines (Fig. 5d, Supplementary Fig. S11). To identify a potential connection between FGFR- and YAP-1 mediated signals, which could impact the observed sprouting behaviour, we performed western blots (Supplementary Fig. S12). Our data showed no significant changes in phospho-YAP1 and the YAP1 target gene Cysteine-rich angiogenic inducer 61 (CYR61), when FGFR-mediated signals were stimulated with FGF2 or inhibited with erdafitinib. However, as expected, we could detect a respective increase or decrease in phosphorylation of ERK, which is downstream of FGFR (Supplementary Fig. S12).

### Targeting the FGF/R axis reduces invasive SCLC behaviour in vivo

**Fig. 6:**
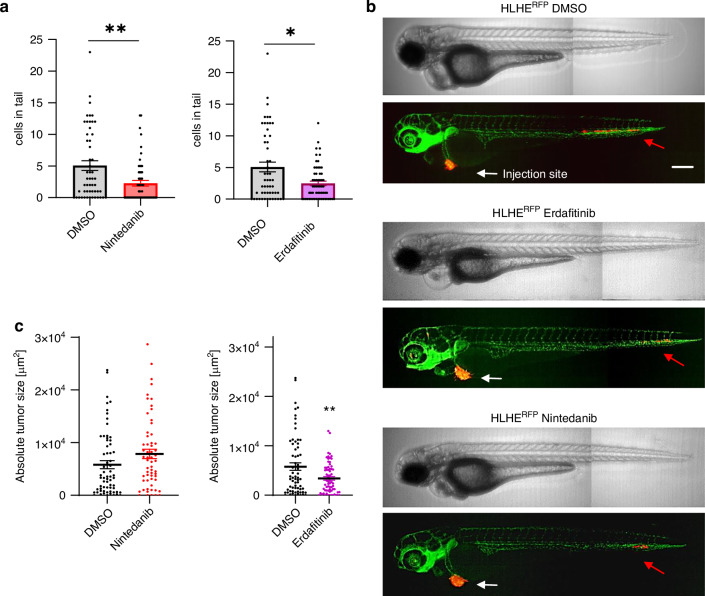
FGFR inhibition reduces the number of extravasated tumour cells in vivo. **a** Total cell number in the tail of zebrafish larvae and **b** absolute tumour size were determined after 24 h exposure to nintedanib and erdafitinib at a concentration of 10 µM. Mann-Whitney test. *p < 0.05, **p < 0.01. **c** Representative images of fli1a: EGFP transgenic zebrafish injected with RFP-expressing HLHE cells, after 24 h treatment with DMSO, nintedanib (10 µM) or erdafitinib (10 µM). White arrows indicate the injection sites, red arrows indicate tumour cells in the tail. Scale bar: 250 µm.

Finally, to study the impact of FGFR inhibition on SCLC invasion in vivo, we performed zebrafish xenografts again. RFP-expressing HLHE cells were xenotransplanted into the perivitelline space of zebrafish larvae and cell migration was determined following 48 h treatment with 10 µM nintedanib or erdafitinib, starting from 1 dpi. Upon exposure with either of the drugs, we found a significant decrease in the total number of cells that migrated into the tail at 3 dpi, confirming our in vitro results that inhibiting the FGF/R axis significantly reduces the invasive potential of SCLC cells (Fig. 6a, b). Furthermore, erdafitinib significantly reduced the absolute tumour sizes, however, interestingly, nintedanib had no effect (Fig. 6c).

Taken together, our data show that the invasive potential of SCLC is subtype specific and preventable by FGFR inhibition, thereby providing new insights into the molecular mechanisms underlying SCLC metastasis.

## Discussion

Therapeutic innovation in SCLC has been hindered by insufficient tissue sampling, due the inevitable metastatic spread seen in almost every patient. The metastatic burden is the primary driver of rapid disease progression and remains one the greatest hurdles in fighting this hard-to-treat disease. In the past years, preclinical and translational research has provided evidence of underlying molecular differences within SCLC. Initially, the four transcriptional factors ASCL1, NEUROD1, POU2F3 and YAP1 were reported to represent markers defining specific subtypes. Recently, an inflamed SCLC subtype with mesenchymal features was described since YAP1 could not be verified to be an independent subtype-defining marker in clinical SCLC specimens [5, 21]. A robust molecular classification system for SCLC remains to be established.

The interplay between subtype heterogeneity and metastatic potential is crucial in understanding SCLC progression. It has been shown that, metastatic SCLC tumour cells display distinct organotropism, initially spreading to the liver and adrenal glands before advancing to the brain, bone and pericardium in later stages [3]. Of note, patients with extrathoracic metastases have evidently reduced overall survival irrespective of the location of the metastatic site in comparison to patient without distant organ metastases at diagnosis [22]. Accumulating evidence underlines that SCLC neoplasms commonly display intratumoral heterogeneity, namely a composition of cancerous cells with subtype-defining NE and non-NE properties [23]. In line with this, YAP1 expression is commonly known to be inversely correlated with numerous NE markers [24]. Interplay between intratumoral cells with different genetic backgrounds has been proposed to be crucial for the initial steps of metastatic spread, including the process of epithelial to mesenchymal transition (EMT) [25].

While the cell adhesion molecule E-cadherin (CDH1) is increased in early lesions, it is tightly downregulated in metastatic SCLC [26]. High VIM expression denotes a more mesenchymal state, however, VIM expression was generally low in a study investigating circulating tumour cells of SCLC patients [27]. Whereas, VIM expression was recently shown to be almost exclusively restricted to inflamed SCLC primary tumours of surgically resected patients, YAP1 is also co-expressed in other SCLC subtypes. VIM-positive SCLCs displayed a significantly worse overall survival compared to the two NE-subtypes, SCLC-A and SCLC-N. SCLC-A tumours furthermore displayed a beneficial response to adjuvant chemotherapy and reported the opposed observation of VIM-high tumours, which is in line with the occurrence of VIM-expressing cells after cisplatin-resistance based on a circulating tumour cell-derived xenograft model [28].

In the current study, we report an association between increased sprouting, metastatic potential, and elevated levels of key EMT markers, including VIM, TWIST1, SNAI1 and MMP1. These molecules are well known for facilitating metastatic dissemination via tissue remodelling, cellular migration and invasion. Moreover, all of these proteins are linked to the FGF/FGFR axis by either being regulated by this signalling cascade or mediating the expression of various growth factors and their receptors [29, 30]. Functionally validating an association between the FGFR-mediated signals and invasive behaviour, our data show a significant increase in sprouting when cells are stimulated with FGF2. Fittingly, we have previously reported similar observations in pleural mesothelioma [10].

The distinction between ‘sprouters’ and ‘non-sprouters’ in our SCLC cell models also corresponds to YAP1 vs. non-YAP1 subtype differentiation. This is in line with our previously published study which showed that YAP1-driven SCLC cells tend to express markers associated with mesenchymal-like behaviour, leading to a more aggressive phenotype [6] and may also contribute to the fact that patients with YAP1-high tumours tend to have worse overall survival outcomes [31]. To address whether the observed phenotypic differences are intrinsically linked to YAP1, we investigated if modulation of YAP1 activity directly affects invasive sprouting and showed that neither inhibition nor activation of YAP1 transcriptional activity via K-975 and XMU-MP-1, respectively, significantly altered the sprouting ability of our cell models. Furthermore, YAP1 transcriptional activity was not influenced by activation or inhibition of FGFR-mediated signals by FGF2 and erdafitinib, respectively. These findings suggest that, despite the different YAP1 status, invasive sprouting itself appears to be independent of YAP1 signalling, supporting the dominant role of FGFR-mediated signals in driving aggressive behaviour.

Furthermore, our data show overexpression of the FGF/R axis in YAP1-driven ‘sprouter’ cells. These powerful signalling molecules physiologically regulate embryonic development and adult tissue homoeostasis [32]. In many cancer types including lung cancer and pleural mesothelioma, aberrant FGFR signalling has been proposed to drive cancer development, progression and metastasis [33, 34]. Amplification of the FGFR1 gene has been detected in defined subpopulations of both SCLC and NSCLC and has been proven to be a driving oncogene [35–37]. The FGF axis seems to play a central role in lung cancer metastasis, which was also reported in context with SRY-box 2 (SOX2). In FGFR1-amplified lung cancer including a SCLC cell line, activation by its specific ligand, FGF2 promoted cell proliferation and EMT, which resulted in invasion. This FGFR activation process has been shown to be mediated via increased expression of SOX2 by downstream phosphorylated ERK1/2 [38]. FGFR1 protein expression determined by immunohistochemistry was shown to significantly correlate with FGFR1 gene copy number and mRNA levels in a broad panel of SCLC specimens [39].

Another previously reported potential mechanism mediating intratumoral heterogeneity and influencing the metastatic potential is through the secretion of FGF2 by non-NE SCLC cells [40]. In our study, besides HGF, we found higher amounts of FGF2, FGF4 and FGF6 in the supernatant of sprouters compared to non-sprouters. A recent analysis aiming to identify mutated gene networks in SCLC revealed that mutations in HGF and FGFR2, among others, are highly associated with prolonged survival [41]. Also, intratumoral heterogeneity is occasionally associated with the transdifferentiation from lung adenocarcinoma into SCLC. In context of the FGF/R axis, FGF9 upregulation has been demonstrated to promote transdifferentiation via induction of NE differentiation. Consequently, the therapeutic approach using a pan-FGFR inhibitor fexagratinib resulted in effective growth inhibition of transformed SCLC-like tumours in vivo [42]. Similarly, the in vitro and in vivo efficacy of the pan-FGFR inhibitor LY2874455 has been linked to the FGFR-PEA3 signalling axis. Polyomavirus enhancer activator 3 (PEA3) represents an important member of the Ets transcription factor family. Delayed disease progression following combination chemotherapy was due to the specific inhibition of the FGFR substrate 2 (FRS2) leading to downstream inactivation of PI3K-Akt and MAPK signalling [43].

Fittingly, we identified that inhibition of the FGF axis via erdafitinib and nintedanib had the most potent effects in prevention of invasive sprouting in our in vitro and in vivo models. Nintedanib, a multi-tyrosine kinase inhibitor targeting FGFR, VEGFR and PDGFR, has been extensively investigated as a potential cancer therapy in recent years. The European Medicines Agency approved this agent as a second-line therapy option after chemotherapy failure in progressive lung adenocarcinoma based on encouraging trial results [44, 45]. Recent research outcomes also reported promising results for efficacy and tolerable primary endpoints of nintedanib in combination with carboplatin and etoposide in SCLC patients with comorbid idiopathic pulmonary fibrosis [46]. Notably, erdafitinib, a selective pan-FGFR inhibitor (FGFR1-4), has shown clinical benefit and received FDA approval for treatment of advanced or metastatic urothelial carcinoma harbouring FGFR2 or FGFR3 alterations [47]. Ongoing clinical trials are currently evaluating erdafitinib in other solid tumours with FGFR alterations, including gastrointestinal cancers (NCT02699606) and NSCLC (NCT03827850), further underscoring the broader therapeutic potential of FGFR inhibition.

Taken together, this study demonstrated that the FGF/R signalling cascade is an essential driver of SCLC aggressiveness, particularly in YAP1-driven SCLC. In vitro and in vivo invasion was significantly inhibited using FGFR inhibition. Lower levels of FGF-related markers in non-sprouter cell lines support the hypothesis that sprouters rely more heavily on FGF/FGFR signalling for metastatic spread, which we have functionally confirmed. SCLC disease outcome is significantly negatively influenced by the inevitable occurrence of distant metastases. Hence, the molecular drivers of metastatic dissemination need to be adequately characterised. Inhibition of the FGF/R axis might serve as a promising approach to target metastasis formation in certain SCLC subtypes and warrants further investigation.

## Supplementary information

Supplementary Figures

Supplementary Table 1

Supplementary Table 2

Supplementary Table 3

Supplementary Table 4

## Data Availability

Data were generated by the authors and available on reasonable request.
