# Supplementary material for: Fibroblast growth factor signals drive the metastatic behavior in small cell lung cancer
Source: Br J Cancer. 2025 Dec 13;134(4):543–54. doi: 10.1038/s41416-025-03276-y (PMC12859120; doi:10.1038/s41416-025-03276-y)
Supplement: Supplementary file 1 — Supplementary Figures [file 41416_2025_3276_MOESM1_ESM.pdf]

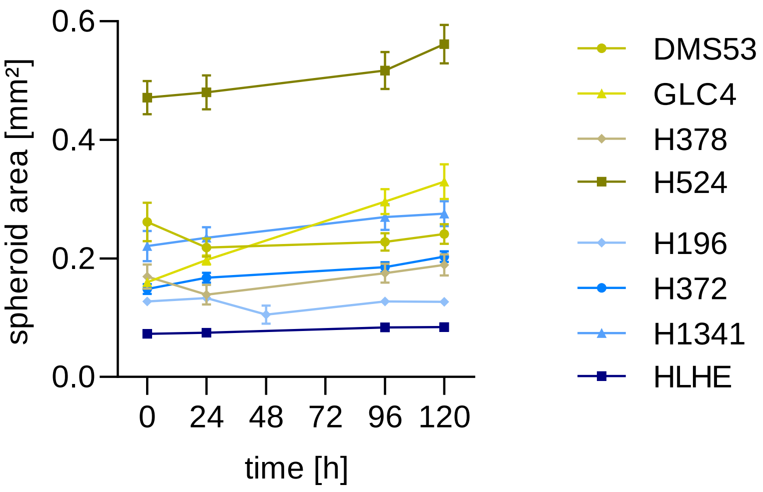

Supplementary Figure S1: Non-sprouter SCLC cell lines generate bigger spheroids in 3D. Spheroid area over time of spheroid-forming SCLC cell lines grouped into sprouter and non-sprouter cell lines. Data is shown as mean ± SEM of at least 15 individual spheroids per cell line.

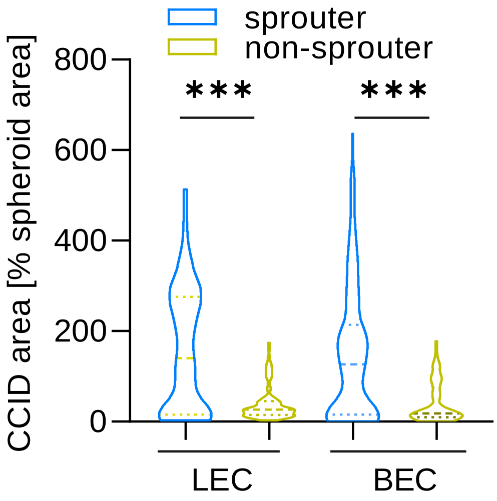

Supplementary Figure S2: Sprouter cells form larger CCIDs. Groupwise comparison between sprouter (blue; HLHE, H372, H1341, H196) and non-sprouter (ochre; H524, GLC4, DMS53, H378) cell lines by means of BEC and LEC displacement. ANOVA and Kruskal-Wallis test. ***p<0.001.

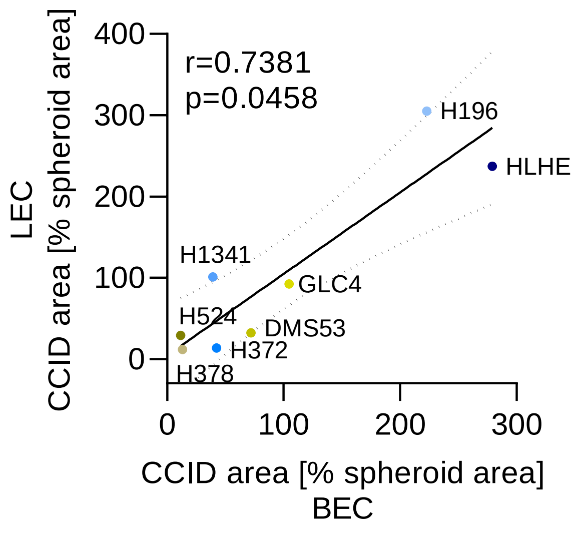

Supplementary Figure S3: CCID formation is similar in BECs and LECs. Spearman correlation analysis between circular chemorepellent-induced defects (CCID) in LEC (y-axis) and BEC (x-axis) cells caused by SCLC spheroids after 120 h.

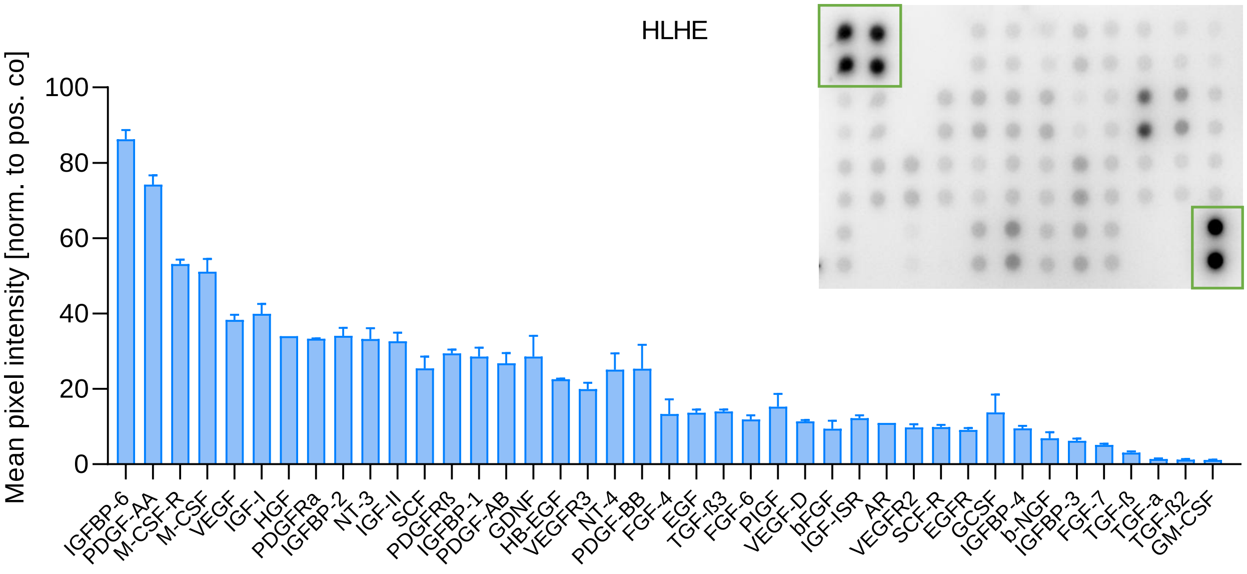

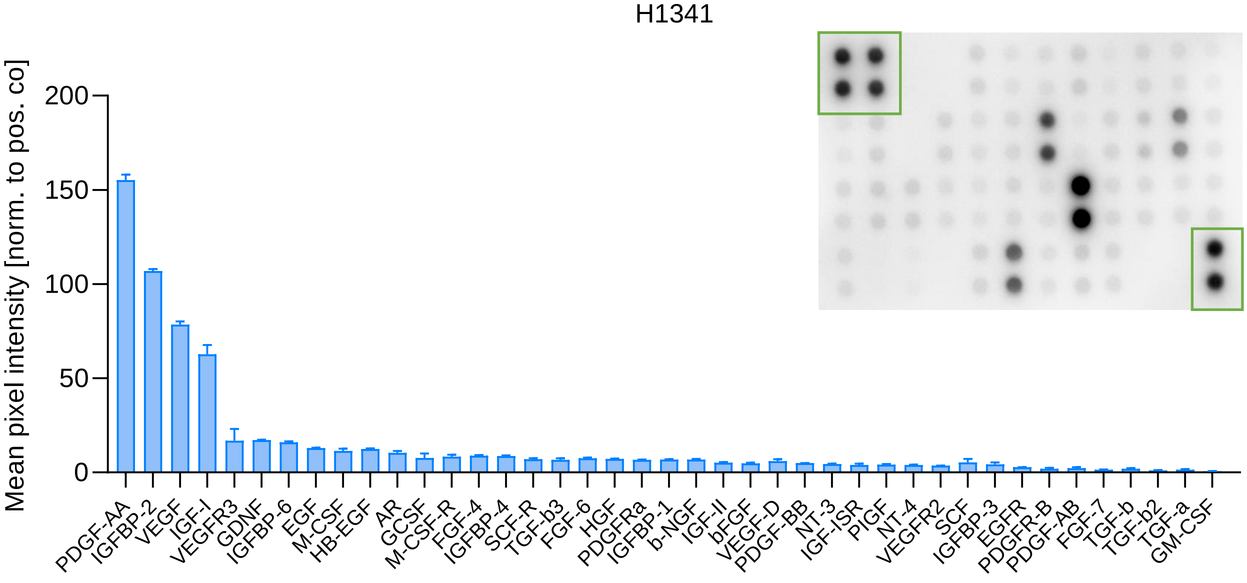

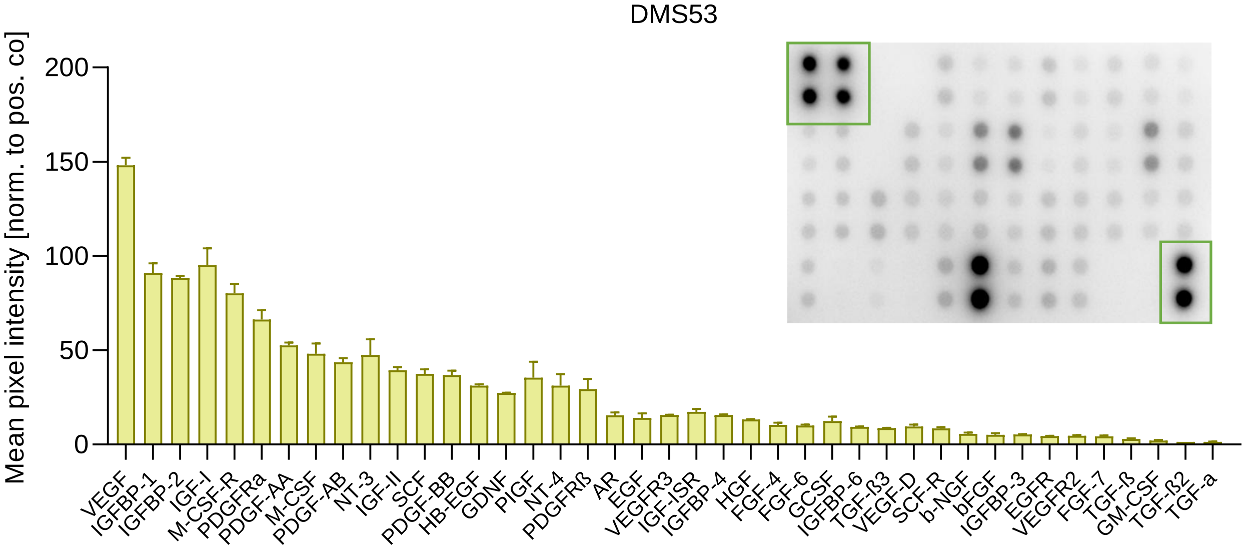

Ernhofer et al. Supplementary Figure S4

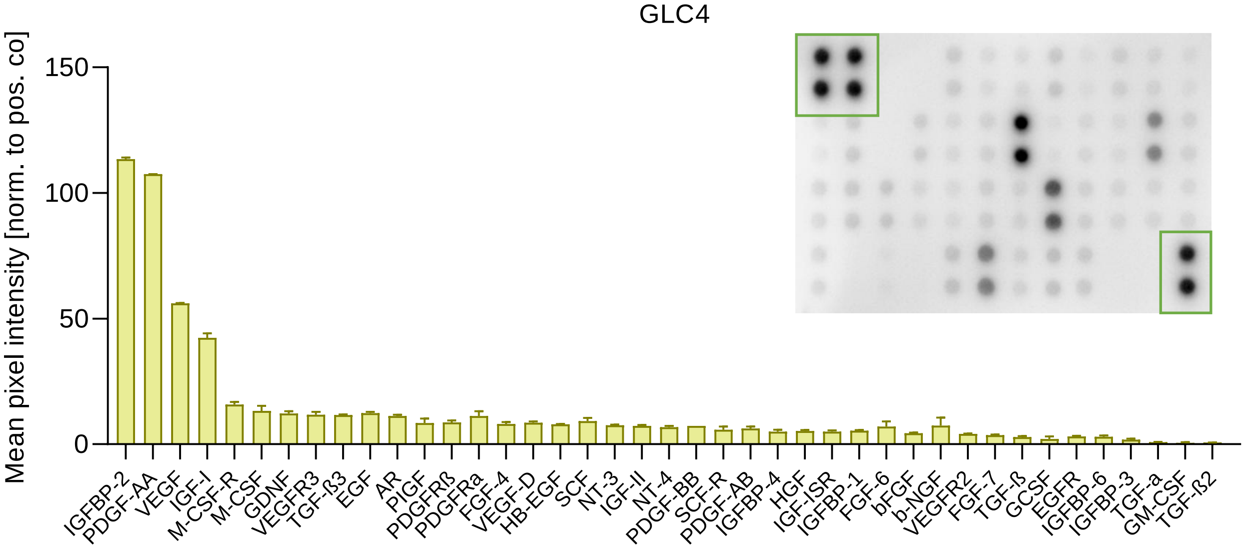

Supplementary Figure S4: Growth factor secretion varies between two SCLC cell lines representative of the sprouter and non-sprouter groups. Individual data of secreted growth factors of HLHE and H1341 (sprouter; blue) and DMS53 and GLC4 (non-sprouter; ochre). SCLC cells were incubated in serum-free medium for 24 h and shed growth factors in the supernatant were measured. Data is shown as mean ± SEM and positive controls of each blot was used as reference (green rectangles).

APICAL_JUNCTION

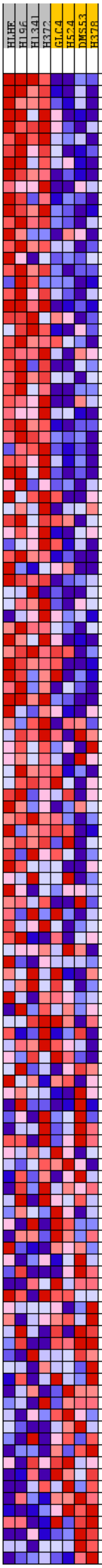

SampleName		
ITGA3	ITGA3	integrin subunit alpha 3 [Source:HGNC Symbol;Acc:HGNC:6139]
EGFR	EGFR	epidermal growth factor receptor [Source:HGNC Symbol;Acc:HGNC:3236]
TSPAN4	TSPAN4	tetraspanin 4 [Source:HGNC Symbol;Acc:HGNC:11859]
SORBS3	SORBS3	sorbin and SH3 domain containing 3 [Source:HGNC Symbol;Acc:HGNC:30907]
SYK	SYK	spleen associated tyrosine kinase [Source:HGNC Symbol;Acc:HGNC:11491]
ZYX	ZYX	zyxin [Source:HGNC Symbol;Acc:HGNC:13200]
CD99	CD99	CD99 molecule (Xg blood group) [Source:HGNC Symbol;Acc:HGNC:7082]
FLNC	FLNC	filamin C [Source:HGNC Symbol;Acc:HGNC:3756]
RRAS	RRAS	RAS related [Source:HGNC Symbol;Acc:HGNC:10447]
ICAM1	ICAM1	intercellular adhesion molecule 1 [Source:HGNC Symbol;Acc:HGNC:5344]
PARVA	PARVA	parvin alpha [Source:HGNC Symbol;Acc:HGNC:14652]
MSN	MSN	moesin [Source:HGNC Symbol;Acc:HGNC:7373]
MYH9	MYH9	myosin heavy chain 9 [Source:HGNC Symbol;Acc:HGNC:7579]
THY1	THY1	Thy-1 cell surface antigen [Source:HGNC Symbol;Acc:HGNC:11801]
ACTN1	ACTN1	actinin alpha 1 [Source:HGNC Symbol;Acc:HGNC:163]
BMP1	BMP1	bone morphogenetic protein 1 [Source:HGNC Symbol;Acc:HGNC:1067]
CNN2	CNN2	calponin 2 [Source:HGNC Symbol;Acc:HGNC:2156]
ACTA1	ACTA1	actin alpha 1, skeletal muscle [Source:HGNC Symbol;Acc:HGNC:129]
MYL12B	MYL12B	myosin light chain 12B [Source:HGNC Symbol;Acc:HGNC:29827]
ACTN4	ACTN4	actinin alpha 4 [Source:HGNC Symbol;Acc:HGNC:166]
NF1	NF1	neurofibromin 1 [Source:HGNC Symbol;Acc:HGNC:7765]
DLG1	DLG1	discs large MAGUK scaffold protein 1 [Source:HGNC Symbol;Acc:HGNC:2900]
MAP3K20	MAP3K20	mitogen-activated protein kinase kinase kinase 20 [Source:HGNC Symbol;Acc:HGNC:17797]
ADAM9	ADAM9	ADAM metallopeptidase domain 9 [Source:HGNC Symbol;Acc:HGNC:216]
ITGB1	ITGB1	integrin subunit beta 1 [Source:HGNC Symbol;Acc:HGNC:6153]
MPZL1	MPZL1	myelin protein zero like 1 [Source:HGNC Symbol;Acc:HGNC:7226]
VASP	VASP	vasodilator stimulated phosphoprotein [Source:HGNC Symbol;Acc:HGNC:12652]
SHC1	SHC1	SHC adaptor protein 1 [Source:HGNC Symbol;Acc:HGNC:10840]
NF2	NF2	NF2, moesin-ezrin-radixin like (MERLIN) tumor suppressor [Source:HGNC Symbol;Acc:HGNC:7773]
CAP1	CAP1	cyclase associated actin cytoskeleton regulatory protein 1 [Source:HGNC Symbol;Acc:HGNC:20040]
CD276	CD276	CD276 molecule [Source:HGNC Symbol;Acc:HGNC:19137]
GNAI1	GNAI1	G protein subunit alpha i1 [Source:HGNC Symbol;Acc:HGNC:4384]
VCAN	VCAN	versican [Source:HGNC Symbol;Acc:HGNC:2464]
ARPC2	ARPC2	actin related protein 2/3 complex subunit 2 [Source:HGNC Symbol;Acc:HGNC:705]
NECTIN3	NECTIN3	nectin cell adhesion molecule 3 [Source:HGNC Symbol;Acc:HGNC:17664]
NECTIN2	NECTIN2	nectin cell adhesion molecule 2 [Source:HGNC Symbol;Acc:HGNC:9707]
IRS1	IRS1	insulin receptor substrate 1 [Source:HGNC Symbol;Acc:HGNC:6125]
ARHGEF6	ARHGEF6	Rac/Cdc42 guanine nucleotide exchange factor 6 [Source:HGNC Symbol;Acc:HGNC:685]
B4GALT1	B4GALT1	beta-1,4-galactosyltransferase 1 [Source:HGNC Symbol;Acc:HGNC:924]
SHROOM2	SHROOM2	shroom family member 2 [Source:HGNC Symbol;Acc:HGNC:630]
ACTG2	ACTG2	actin gamma 2, smooth muscle [Source:HGNC Symbol;Acc:HGNC:145]
CD274	CD274	CD274 molecule [Source:HGNC Symbol;Acc:HGNC:17635]
GNAI2	GNAI2	G protein subunit alpha i2 [Source:HGNC Symbol;Acc:HGNC:4385]
CDH3	CDH3	cadherin 3 [Source:HGNC Symbol;Acc:HGNC:1762]
ADAM15	ADAM15	ADAM metallopeptidase domain 15 [Source:HGNC Symbol;Acc:HGNC:193]
LAMA3	LAMA3	laminin subunit alpha 3 [Source:HGNC Symbol;Acc:HGNC:6483]
MAPK14	MAPK14	mitogen-activated protein kinase 14 [Source:HGNC Symbol;Acc:HGNC:6876]
LIMA1	LIMA1	LIM domain and actin binding 1 [Source:HGNC Symbol;Acc:HGNC:24636]
YWHAH	YWHAH	tyrosine 3-monooxygenase/tryptophan 5-monooxygenase activation protein eta [Source:HGNC Symbol;Acc:HGNC:12853]
PFN1	PFN1	profilin 1 [Source:HGNC Symbol;Acc:HGNC:8881]
MAP4K2	MAP4K2	mitogen-activated protein kinase kinase kinase kinase 2 [Source:HGNC Symbol;Acc:HGNC:6864]
EXOC4	EXOC4	exocyst complex component 4 [Source:HGNC Symbol;Acc:HGNC:30389]
ACTG1	ACTG1	actin gamma 1 [Source:HGNC Symbol;Acc:HGNC:144]
ITGA2	ITGA2	integrin subunit alpha 2 [Source:HGNC Symbol;Acc:HGNC:6137]
TJP1	TJP1	tight junction protein 1 [Source:HGNC Symbol;Acc:HGNC:11827]
SLIT2	SLIT2	slit guidance ligand 2 [Source:HGNC Symbol;Acc:HGNC:11086]
TSC1	TSC1	TSC complex subunit 1 [Source:HGNC Symbol;Acc:HGNC:12362]
INPPL1	INPPL1	inositol polyphosphate phosphatase like 1 [Source:HGNC Symbol;Acc:HGNC:6080]
WASL	WASL	WASP like actin nucleation promoting factor [Source:HGNC Symbol;Acc:HGNC:12735]
FSCN1	FSCN1	fascin actin-bundling protein 1 [Source:HGNC Symbol;Acc:HGNC:11148]
SGCE	SGCE	sarcoglycan epsilon [Source:HGNC Symbol;Acc:HGNC:10808]
CTNNA1	CTNNA1	catenin alpha 1 [Source:HGNC Symbol;Acc:HGNC:2509]
VCL	VCL	vinculin [Source:HGNC Symbol;Acc:HGNC:12665]
GTF2F1	GTF2F1	general transcription factor IIF subunit 1 [Source:HGNC Symbol;Acc:HGNC:4652]
AKT2	AKT2	AKT serine/threonine kinase 2 [Source:HGNC Symbol;Acc:HGNC:392]
PTEN	PTEN	phosphatase and tensin homolog [Source:HGNC Symbol;Acc:HGNC:9588]
PTK2	PTK2	protein tyrosine kinase 2 [Source:HGNC Symbol;Acc:HGNC:9611]
ACTN2	ACTN2	actinin alpha 2 [Source:HGNC Symbol;Acc:HGNC:164]
FBN1	FBN1	fibrillin 1 [Source:HGNC Symbol;Acc:HGNC:3603]
HRAS	HRAS	HRas proto-oncogene, GTPase [Source:HGNC Symbol;Acc:HGNC:5173]
DSC1	DSC1	desmocollin 1 [Source:HGNC Symbol;Acc:HGNC:3035]
ICAM5	ICAM5	intercellular adhesion molecule 5 [Source:HGNC Symbol;Acc:HGNC:5348]
GRB7	GRB7	growth factor receptor bound protein 7 [Source:HGNC Symbol;Acc:HGNC:4567]
ITGA9	ITGA9	integrin subunit alpha 9 [Source:HGNC Symbol;Acc:HGNC:6145]
RASA1	RASA1	RAS p21 protein activator 1 [Source:HGNC Symbol;Acc:HGNC:9871]
IKBKG	IKBKG	inhibitor of nuclear factor kappa B kinase regulatory subunit gamma [Source:HGNC Symbol;Acc:HGNC:5961]
CLDN11	CLDN11	claudin 11 [Source:HGNC Symbol;Acc:HGNC:8514]
RAC2	RAC2	Rac family small GTPase 2 [Source:HGNC Symbol;Acc:HGNC:9802]
THBS3	THBS3	thrombospondin 3 [Source:HGNC Symbol;Acc:HGNC:11787]
ACTN3	ACTN3	actinin alpha 3 [Source:HGNC Symbol;Acc:HGNC:165]
STX4	STX4	syntaxin 4 [Source:HGNC Symbol;Acc:HGNC:11439]
MYH10	MYH10	myosin heavy chain 10 [Source:HGNC Symbol;Acc:HGNC:7568]
TUBG1	TUBG1	tubulin gamma 1 [Source:HGNC Symbol;Acc:HGNC:12417]
NEGR1	NEGR1	neuronal growth regulator 1 [Source:HGNC Symbol;Acc:HGNC:17302]
CTNND1	CTNND1	catenin delta 1 [Source:HGNC Symbol;Acc:HGNC:2515]
RSU1	RSU1	Ras suppressor protein 1 [Source:HGNC Symbol;Acc:HGNC:10464]
RHOF	RHOF	ras homolog family member F, filopodia associated [Source:HGNC Symbol;Acc:HGNC:15703]
CRAT	CRAT	carnitine O-acetyltransferase [Source:HGNC Symbol;Acc:HGNC:2342]
SYMPK	SYMPK	symplekin scaffold protein [Source:HGNC Symbol;Acc:HGNC:22935]
TNFRSF11B	TNFRSF11B	TNF receptor superfamily member 11b [Source:HGNC Symbol;Acc:HGNC:11909]
AKT3	AKT3	AKT serine/threonine kinase 3 [Source:HGNC Symbol;Acc:HGNC:393]
EPB41L2	EPB41L2	erythrocyte membrane protein band 4.1 like 2 [Source:HGNC Symbol;Acc:HGNC:3379]
CDH11	CDH11	cadherin 11 [Source:HGNC Symbol;Acc:HGNC:1750]
CNTN1	CNTN1	contactin 1 [Source:HGNC Symbol;Acc:HGNC:2171]
PIK3CB	PIK3CB	phosphatidylinositol-4,5-bisphosphate 3-kinase catalytic subunit beta [Source:HGNC Symbol;Acc:HGNC:8976]
MVD	MVD	mevalonate diphosphate decarboxylase [Source:HGNC Symbol;Acc:HGNC:7529]
TGFBI	TGFBI	transforming growth factor beta induced [Source:HGNC Symbol;Acc:HGNC:11771]
PLCG1	PLCG1	phospholipase C gamma 1 [Source:HGNC Symbol;Acc:HGNC:9065]
LAMC2	LAMC2	laminin subunit gamma 2 [Source:HGNC Symbol;Acc:HGNC:6493]
CLDN6	CLDN6	claudin 6 [Source:HGNC Symbol;Acc:HGNC:2048]
NEXN	NEXN	nexilin F-actin binding protein [Source:HGNC Symbol;Acc:HGNC:29557]
TIAL1	TIAL1	TIA1 cytotoxic granule associated RNA binding protein like 1 [Source:HGNC Symbol;Acc:HGNC:11804]
TRO	TRO	trophinin [Source:HGNC Symbol;Acc:HGNC:12326]
CALB2	CALB2	calbindin 2 [Source:HGNC Symbol;Acc:HGNC:1435]
TAOK2	TAOK2	TAO kinase 2 [Source:HGNC Symbol;Acc:HGNC:16835]
CADM2	CADM2	cell adhesion molecule 2 [Source:HGNC Symbol;Acc:HGNC:29849]
JUP	JUP	junction plakoglobin [Source:HGNC Symbol;Acc:HGNC:6207]
NFASC	NFASC	neurofascin [Source:HGNC Symbol;Acc:HGNC:29866]
SRC	SRC	SRC proto-oncogene, non-receptor tyrosine kinase [Source:HGNC Symbol;Acc:HGNC:11283]
PBX2	PBX2	PBX homeobox 2 [Source:HGNC Symbol;Acc:HGNC:8633]
ATP1A3	ATP1A3	ATPase Na+/K+ transporting subunit alpha 3 [Source:HGNC Symbol;Acc:HGNC:801]
ITGB4	ITGB4	integrin subunit beta 4 [Source:HGNC Symbol;Acc:HGNC:6158]
CDK8	CDK8	cyclin dependent kinase 8 [Source:HGNC Symbol;Acc:HGNC:1779]
JAM3	JAM3	junctional adhesion molecule 3 [Source:HGNC Symbol;Acc:HGNC:15532]
GAMT	GAMT	guanidinoacetate N-methyltransferase [Source:HGNC Symbol;Acc:HGNC:4136]
HADH	HADH	hydroxyacyl-CoA dehydrogenase [Source:HGNC Symbol;Acc:HGNC:4799]
BAIAP2	BAIAP2	BAR/IMD domain containing adaptor protein 2 [Source:HGNC Symbol;Acc:HGNC:947]
NECTIN1	NECTIN1	nectin cell adhesion molecule 1 [Source:HGNC Symbol;Acc:HGNC:9706]
VAV2	VAV2	vav guanine nucleotide exchange factor 2 [Source:HGNC Symbol;Acc:HGNC:12658]
EVL	EVL	Enah/Vasp-like [Source:HGNC Symbol;Acc:HGNC:20234]
DHX16	DHX16	DEAH-box helicase 16 [Source:HGNC Symbol;Acc:HGNC:2739]
MDK	MDK	midkine [Source:HGNC Symbol;Acc:HGNC:6972]
CDH1	CDH1	cadherin 1 [Source:HGNC Symbol;Acc:HGNC:1748]
CLDN7	CLDN7	claudin 7 [Source:HGNC Symbol;Acc:HGNC:2049]
MAPK13	MAPK13	mitogen-activated protein kinase 13 [Source:HGNC Symbol;Acc:HGNC:6875]

Ernhofer et al. Supplementary Figure S5

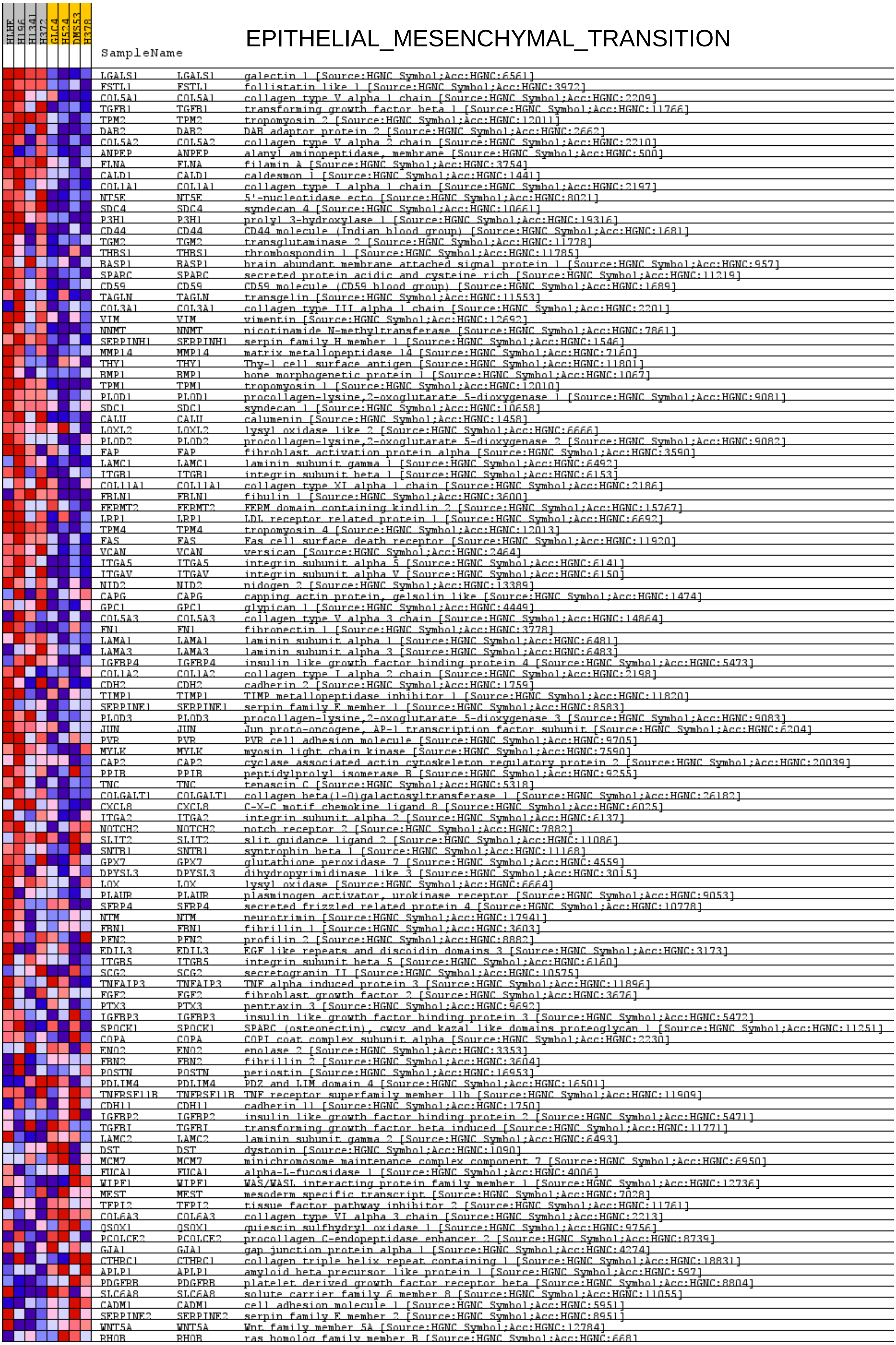

Supplementary Figure S5: Enrichment of apical junction and epithelial-mesenchymal transition in sprouter cell lines. Expression data was generated using the GSEA software (v4.3.2) and the Hallmark dataset.

Ernhofer et al. Supplementary Figure S5 cont.

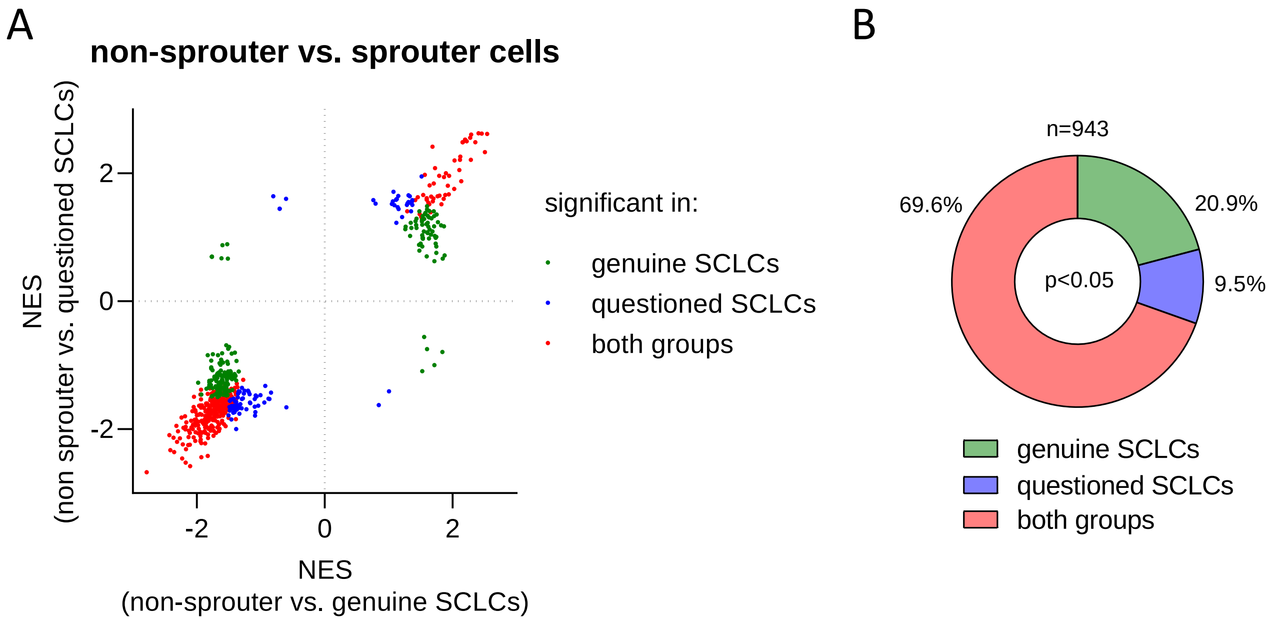

Supplementary Figure S6: Differences and similarities between non-sprouter and genuine or questioned sprouter SCLC lines. (A) Normalized enrichment scores (NES) of pathways from pre-ranked GSEA data was generated using the GSEA software (v4.3.2) and the KEGG, Hallmark and Reactome datasets. Proteomic data from Szeitz et al was analyzed. Non sprouters (DMS53, GCL4, H378, H524) were compared to sprouters - genuine (H372, HLHE) and questioned (H196, H1341) SCLC cells according to Ng. et al. Dots are colored based on significance (nominal $p<0.05$) in one (green, blue) or both (red) comparisons. (B) Percentage of pathways enriched in each or both comparisons.

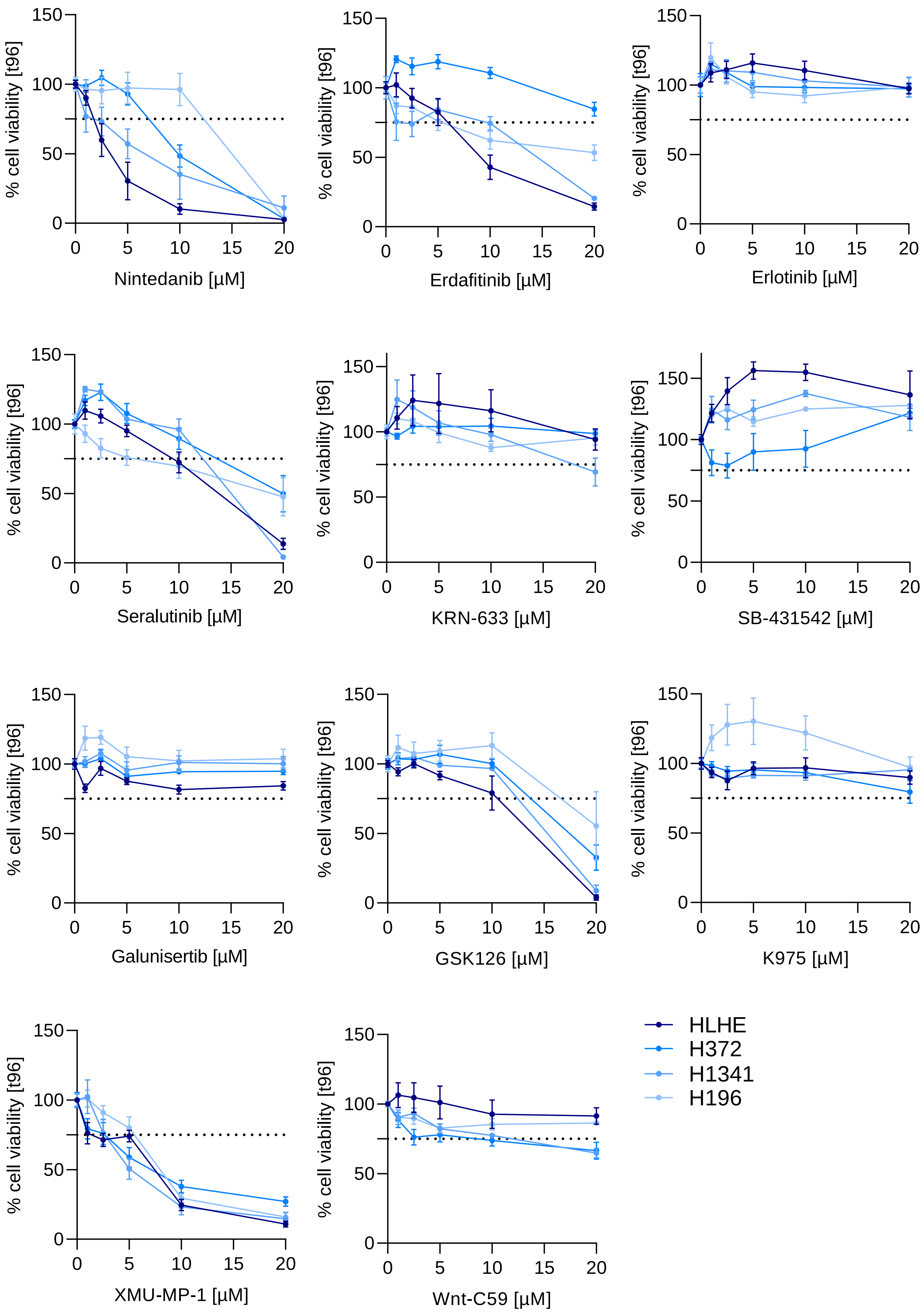

Ernhofer et al. Supplementary Figure S7

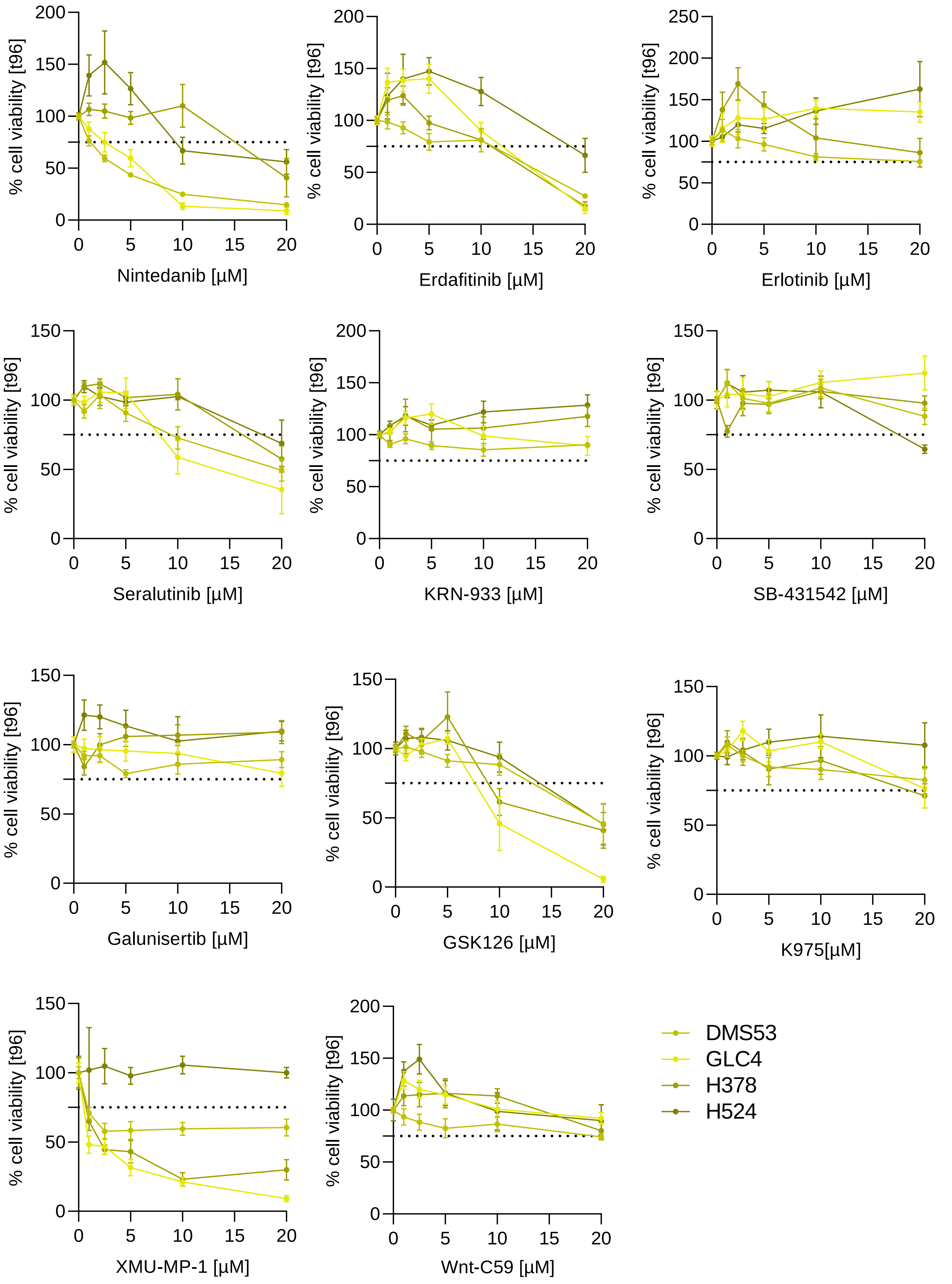

Ernhofer et al. Supplementary Figure S7 cont.

Supplementary Figure S7: Sensitivity to specific pathway inhibition of sprouter and non-sprouter cell lines. Dose response curves from MTT-based cell viability assays testing the inhibitors nintedanib (FGFR, PDGFR, VEGFR), erdafitinib (FGFR1-4), erlotinib (EGFR), seralutinib (PDGFRα,β), KNR-633 (VEGFR1-3), SB-431542 (ALK5/TGFβR), galunisertib (TGFβR), GSK2816126A (GSK126, EZH2), K-975 (YAP1/TAZ-TEAD), XMU-MP-1 (MST1/2) and Wnt-C59 (PORCN) in all cell lines. Sprouter cell lines are shown in blue, non-sprouters in ochre. Data is shown as mean ± SEM compared to DMSO-treated control after 96 h. The dotted line indicates a viability of 75%.

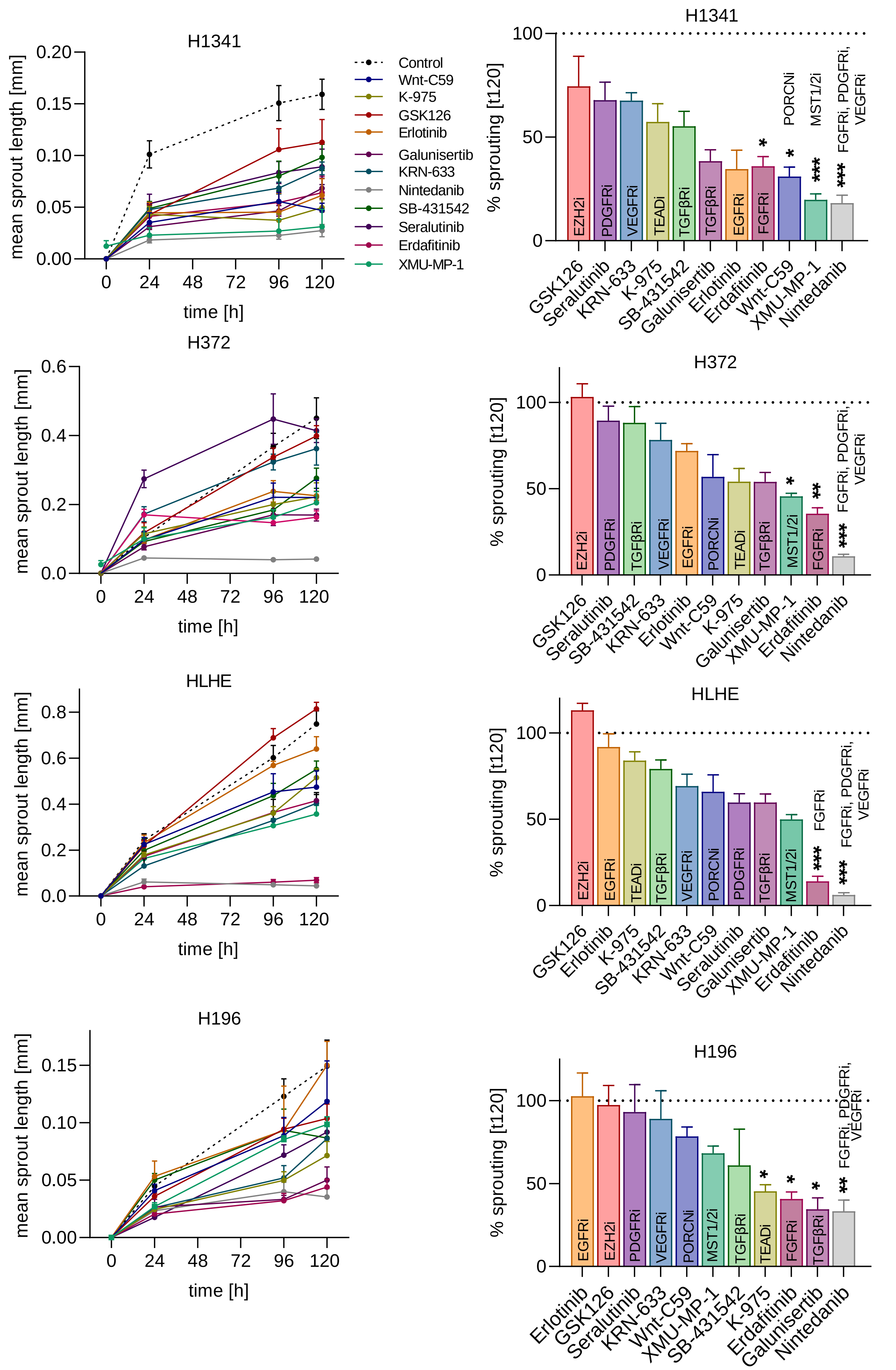

Ernhofer et al. Supplementary Figure S8

Supplementary Figure S8: Drug screening for inhibition of invasive sprouting. Sprouting inhibition based on spheroid sprouting assays using seralutinib (PDGFRα,β), KNR-633 (VEGFR1-3), SB-431542 (ALK5/TGFβR), galunisertib (TGFβR), erdafitinib (FGFR1-4), erlotinib (EGFR), nintedanib (FGFR, PDGFR, VEGFR), GSK2816126A (GSK126, EZH2), K-975 (YAP1/TAZ-TEAD), XMU-MP-1 (MST1/2) and Wnt-C59 (PORCN). Ninetdanib and erdafintinib were used at 0.5 µM, XMU-MP-1 at 1 µM and all other drugs at 10 µM. Mean sprout length over time is shown as mean ± SEM. Bar graphs show sprouting inhibition compared to DMSO-treated control after 120 h (mean ± SEM). Statistical evaluation was performed using one-way ANOVA and DUNN´s multiple comparison test. * $p < 0.05$, ** $p < 0.01$, *** $p < 0.001$.

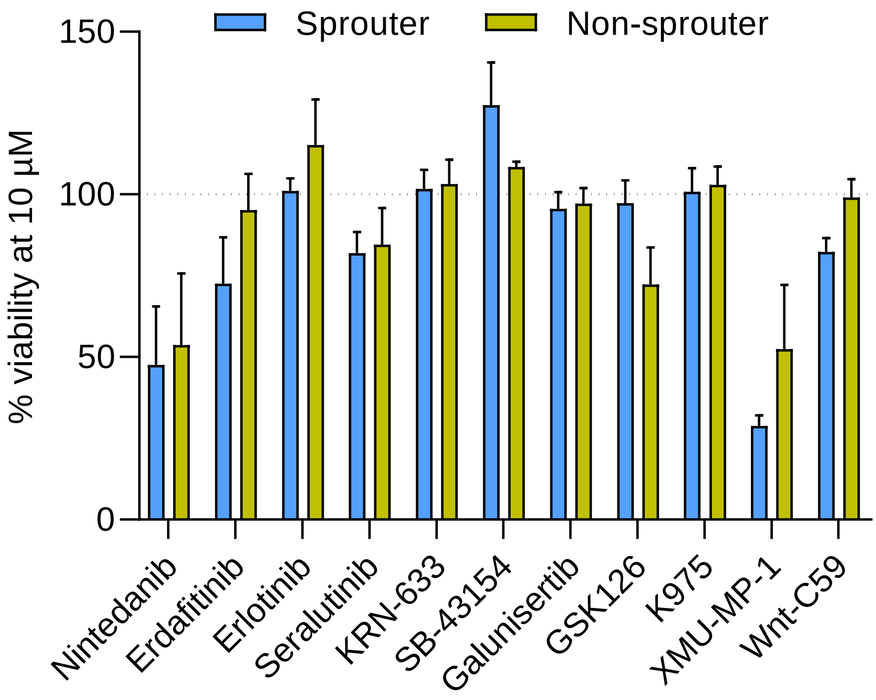

Supplementary Figure S9: Sensitivity to specific pathway inhibition of sprouter and non-sprouter cell lines. Viability at 10 µM, derived from dose response curves from MTT-based cell viability assays testing the inhibitors nintedanib (FGFR, PDGFR, VEGFR), erdafitinib (FGFR1-4), erlotinib (EGFR), seralutinib (PDGFRα,β), KNR-633 (VEGFR1-3), SB-431542 (ALK5/TGFβR), galunisertib (TGFβR), GSK2816126A (GSK126, EZH2), K-975 (YAP1/TAZ-TEAD), XMU-MP-1 (MST1/2) and Wnt-C59 (PORCN) in sprouter (blue) and non-sprouter (ochre) cell lines. Data is shown as as mean ± SEM. Multiple t-tests with Holm-Sidak test. All comparisons: not significant.

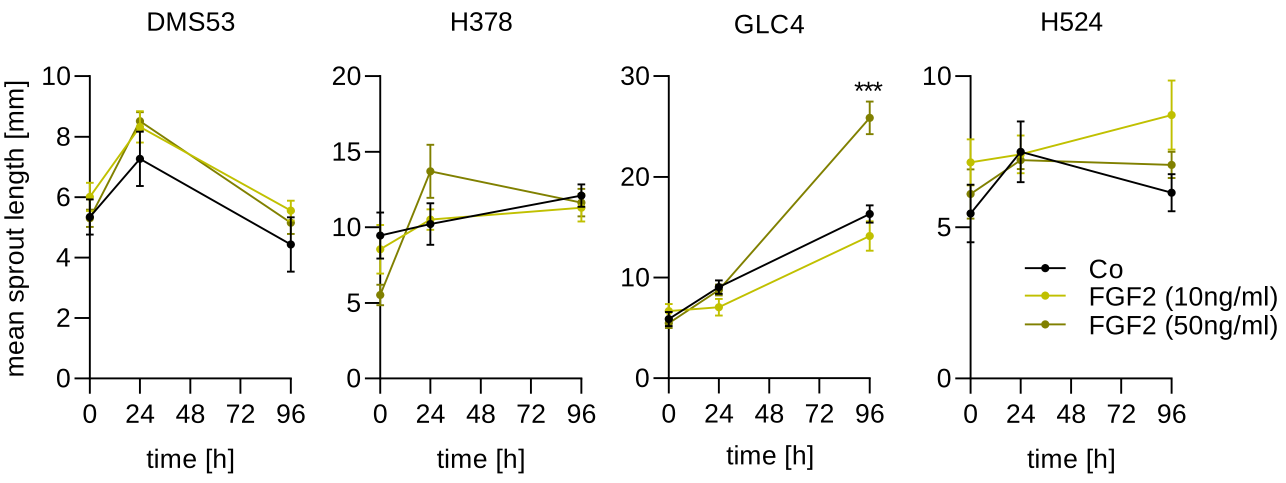

Supplementary Figure S10: Sprouting after stimulation with FGF2. Quantification of sprouting based on spheroid sprouting assays with reduced serum (2.5% FBS). Spheroids were treated with 10 ng/ml and 50 ng/ml recombinant FGF2 for 96 h. Mean sprout length over time is shown as mean ± SEM. Data is shown as mean ± SEM of at least 10 individual spheroids. Statistical evaluation was performed using two-way ANOVA and Tukey´s multiple comparison test. *** $p < 0.001$.

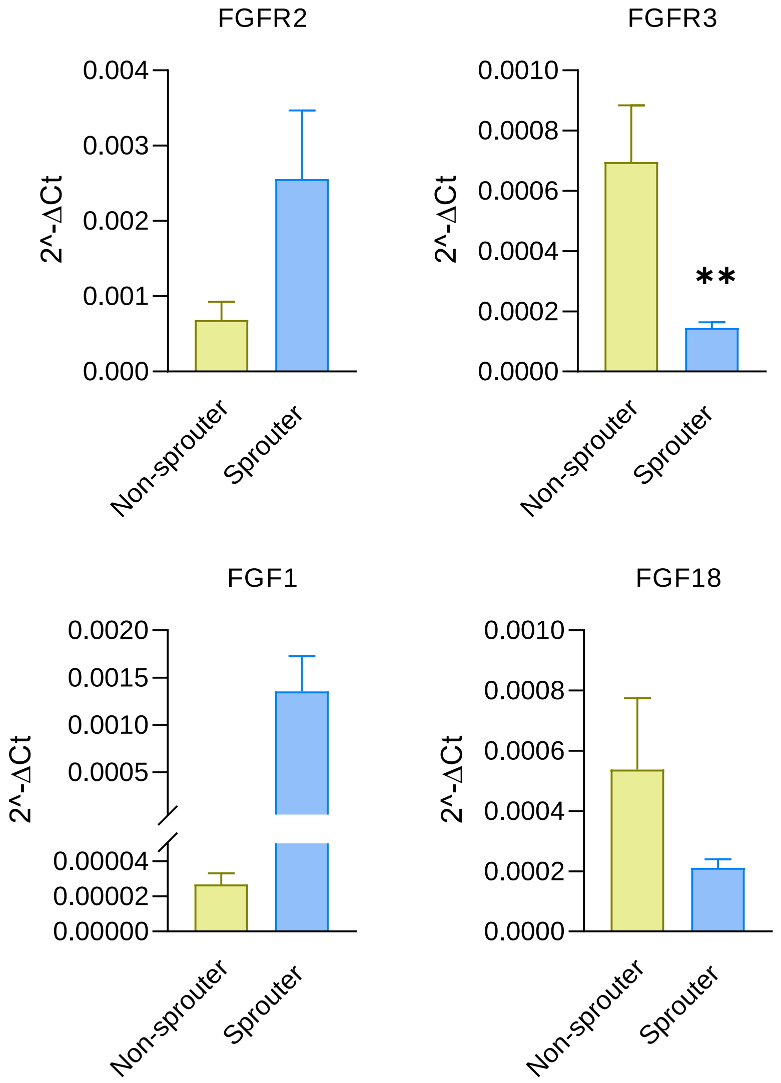

Supplementary Figure S11: Differences in expression of FGF/R family members between sprouters and non-sprouters. RNA expression of FGFRs and FGFs in pooled non-sprouter (ochre) and non-sprouter (blue) cell lines (n=4), determined by qPCR. Data is shown as mean ± SEM. Mann-Whitney test. *p < 0.05, **p < 0.01, ***p < 0.001.

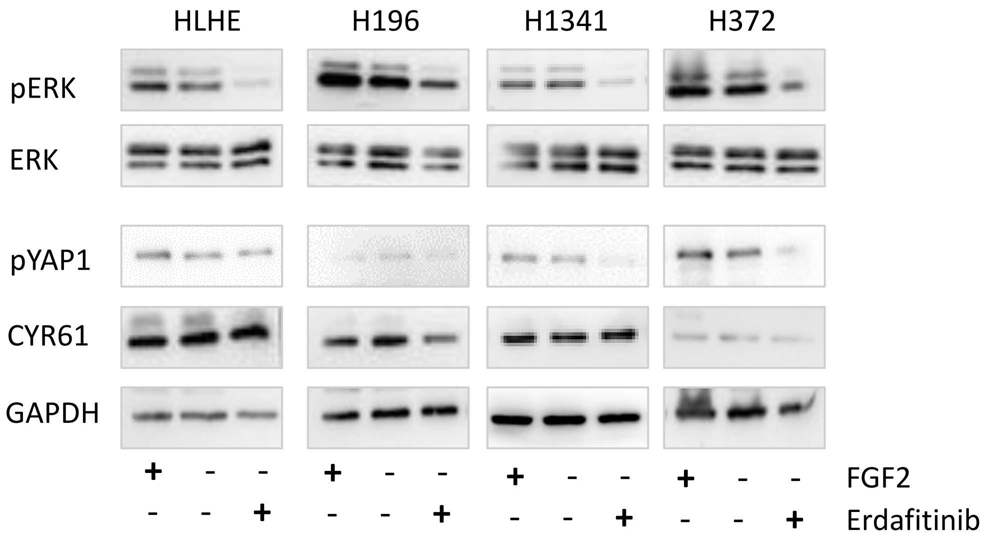

Supplementary Figure S12: Connection between FGFR, MAPK and YAP1 signals. Representative western blot images of sprouter cell lines treated with 10 ng/ml FGF2, 10 µM erdafitinib or vehicle for 24 h. GAPDH was used as loading control.
