# Supplementary material for: Fibroblast growth factor signals drive the metastatic behavior in small cell lung cancer
Source: Br J Cancer. 2025 Dec 13;134(4):543–54. doi: 10.1038/s41416-025-03276-y (PMC12859120; doi:10.1038/s41416-025-03276-y)
Supplement: Supplementary file 2 — Supplementary Table 1 [file 41416_2025_3276_MOESM2_ESM.pdf]

Supplementary Table 1: Cell line characteristics

	Name	Cell line origin	Growth properties	Subtype	Chemotherapy
Sprouter	HLHE	Brain	adherent	YAP1	NA
	H1341*	Cervix	adherent	YAP1	NA
	H372	Bone marrow	adherent	YAP1	NA
	H196*	Pleural effusion	adherent	YAP1	Post-chemo
Non-sprouter	H378	Lung	suspension	ASCL1	Post-chemo
	DMS53	Lung	adherent	ASCL1	Chemo-naïve
	H524	Lymph node	suspension	NEUROD1	Post-chemo
	GLC4	Pleural effusion	semi-adherent	NEUROD1	Chemo-naïve
	H1694	Lung	semi-adherent	NEUROD1	NA
	COR-L311	Lung	suspension	POU2F3	Post-chemo
	H82	Pleural effusion	suspension	NEUROD1	NA
	H1048	Pleural effusion	adherent	YAP1	NA
	H2171	Pleural effusion	suspension	NEUROD1	Chemo+radiation
Endothelial cells	LEC	Lymphatic tissue	adherent	-	-
	BEC	Blood vessel	adherent	-	-

* Cell lines marked with an asterisk have recently been mentioned by Ng. et al (*https://doi.org/10.1158/1078-0432.CCR-23-2360*), questioning the pulmonary origin and small cell nature of these models.

Supplementary Table 2: Names of inhibitors, their molecular targets, source and concentrations used for sprouting assays

Supplementary Table 3: List of differentially expressed proteins between "sprouter" and "non-sprouter" cell lines, derived from proteomics data

Supplementary Table 4: List of significantly dysregulated KEGG pathways between "sprouter" and "non-sprouter" cell lines, derived from 1D annotation enrichment analysis of proteomics data
